# Sanhuang Xiexin Decoction ameliorates lipid disorders in obese mice via inducing browning of white adipose tissue and activating brown adipose tissue

**DOI:** 10.1186/s13020-025-01111-3

**Published:** 2025-05-09

**Authors:** Zhiqi Huang, Ziyang Zhao, Haonan Shi, Runcheng He, Qianqian Feng, Yang Geng, Lei Zhang, Ti Zhang, Zhenteng Yan, Zhanhong Jia, Yanyan Jiang, Jianning Sun, Ang Cai, Shifen Dong, Lin Yang

**Affiliations:** 1 School of Chinese Materia Medica, Beijing University of Chinese Medicine, Beijing, China; 2 Engineering Research Center for Pharmaceutics of Chinese Materia Medica and New Drug Development, Ministry of Education, Beijing, China; 3 Beijing Hospital of Traditional Chinese Medicine, Capital Medical University, Beijing, China; 4 Department of Biomedical and Pharmaceutical Sciences, College of Pharmacy, University of Rhode Island, Kingston, RI USA

**Keywords:** Sanhuang Xiexin Decoction, Obesity, HIB-1B brown adipocyte, 3T3-L1 white adipocyte, Adipose plasticity

## Abstract

**Background:**

Obesity has become a critical public health challenge worldwide. Prioritizing prevention and tackling root causes—rather than merely managing symptoms—is critical to curbing this pandemic. Strategies that activate and expand brown adipose tissue and beige adipose tissue increase energy expenditure in animal models and offer therapeutic promise to treat obesity. Sanhuang Xiexin Decoction (SHX) is a well-known traditional Chinese medicine that possesses several beneficial effects, including anti-inflammatory and anti-atherosclerotic properties. This study aims to investigate whether SHX can alleviate obesity by promoting the browning process in adipose tissue.

**Methods:**

UPLC MS/MS was used to detect the constituents of SHX extraction, as well as the absorbed components of SHX in rat plasma. In vivo, C57BL/6 J mice were fed with 60% calorie high-fat diet for 8 weeks to induce obesity. 3T3-L1 white adipocyte and HIB-1B brown adipocyte were cultured. Parameters of body weight, food intake, Lee’s index, skin temperature, adipose tissue mass, and blood glucose and lipids of mice were detected. The histological features of BAT and iWAT were observed by H&E staining, and the protein expression of UCP1 in adipose tissue was detected by immunohistochemistry method, and the UCP1 protein expression of 3T3-L1 and HIB-1B cells was tested using immunofluorescence staining. Gene expression of browning markers (e.g. *Ucp1*, *Pgc1α*, *Prdm16*, *Cidea*, *Cd137*, *Tbx1*, and *Tmem26*), fatty acid oxidation factors (e.g. *Cpt-1β*, *Cyto-c*, and *Fatp1*) and mitochondrial biogenic transcription factors (e.g. *Nrf1*, *Nrf2*, and *Tfam*) of adipose tissue, 3T3-L1 and HIB-1B cells was detected using qRT-PCR method.

**Results:**

A total of 58 chemical components of Sanhuang Xiexin Decoction were identified by UPLC-LTQ-Orbitrap-MS^n^ method, which provided the basis for the basic research of SHX pharmacodynamic substances. In vivo, SHX extraction could reduce body weight gain, improve glucose and lipid metabolism, enhance the activity of brown adipose tissue and induce the development of brown-like adipocytes of iWAT in obese mice. Furthermore, SHX extraction improved the gene expression of brown markers including *Ucp1*, *Pgc1α* and *Prdm16*, and mitochondrial biogenic transcription factors including *Nrf1* and *Tfam*, as well as the UCP1 protein levels in white and brown adipocytes.

**Conclusions:**

Our study suggested that Sanhuang Xiexin Decoction could be used as a potential therapeutic candidate for the treatment of obesity and its complications by inducing browning of white adipose tissue and activating brown adipose tissue.

**Supplementary Information:**

The online version contains supplementary material available at 10.1186/s13020-025-01111-3.

## Introduction

Obesity and metabolic syndrome are rapidly increasing worldwide, leading to high raises of various complications and mortality [1]. In 2024, the World Health Organization (WHO) reported that 1 in 8 people in the world were living with obesity, 2.5 billion adults were overweight [2]. In China, it is predicted that 61% of adults will be overweight or obese by 2030 [3]. Developing comprehensive strategies to address obesity and its complications is of great importance to the healthcare community. Obesity is the outcome of an imbalance between food intake and energy expenditure, which causes fat cells to multiply and proliferate. In mammals, both brown adipose tissue (BAT) and white adipose tissue (WAT) contribute to systemic energy homeostasis; however, their distinct anatomical, morphological, and functional properties underscore their complementary roles are quite different [4].

Harnessing BAT activity or promoting WAT health could offer strategies to combat obesity and obesity-related metabolic disorders [5]. BAT, a thermogenic tissue with multilocular lipid droplets and a lot of mitochondria, is extensively vascularized and innervated, which promotes energy metabolism by accelerating the oxidation of fatty acids. BAT generates heat in response to cold exposure due to its unique expression of uncoupling protein 1 (UCP1) [6]. WAT, on the other hand, is a major organ for energy storage and is characterized by unilocular lipid droplets and few mitochondria. Recently, beige or brown adipocytes have been identified as a third type of adipocytes with almost identical physiological functions to brown adipocytes with multilocular lipid droplets and intensive mitochondria. In response to exposure to cold and β-adrenergic receptor agonists, beige adipocytes proliferate in the WAT [7].

Sanhuang Xiexin Decoction, originating from Synopsis of the Golden Chamber, is comprised of *Rhei Radix et Rhizoma*, *Coptidis Rhizoma*, and *Scutellariae Radix*, and demonstrates properties of clearing heat, removing fire, cooling blood, stopping bleeding, etc. This formula has been used for over a thousand years in the treatment of hematemesis and epistaxis, including conditions such as gastric hemorrhage and nasal hemorrhage. It also used to treat diabetes and other metabolic disorders [8].

Previous studies manifested that SHX possesses a wide range of pharmacological effects including anti-inflammation [9], anti oxidation [7], anti-apoptosis [10], anti-atherosclerosis [11], and improvement of insulin resistance [12]. Several main components in SHX (e.g., emodin, baicalein, berberine) have been confirmed to have potential protective effects on lipid metabolism disorders.

Our previous research demonstrated that the emodin could improve the lipidomic disorder of adipose tissue in obese mice by activating brown adipose tissue and stimulating browning of subcutaneous adipose tissue [13]. Baicalein might also drastically lower the body weight, fat mass, and circulating glucose levels of obese rats, which might be related to Akt/GLUT4 pathway [14]. Moreover, berberine has been identified as an activator of AMPK [15], and may regulate the AMPK/SIRT1 pathway to facilitate the remodeling of adipose tissue and thermogenesis [16]. Despite the potential benefits of SHX and its components on lipid metabolism disorders, the exact anti-obesity effects and mechanisms of SHX remain unknown. Therefore, this study investigates the impact of SHX on obesity and its potential mechanisms using in vivo and in vitro experiments.

## Material and methods

### Preparation of Sanhuang Xiexin Decoction extract

*Rhei Radix et Rhizoma* (Beijing, batch NO: 20201125), *Coptidis Rhizoma* (Beijing, batch NO: 200301001) and *Scutellariae Radix* (Beijing, batch NO: 200420002) were obtained from Beijing Tongrentang Co., Ltd. (Beijing, China). Rosiglitazone maleate (purity 99%) and CL 316243 were purchased from Shanghai Yuanye Biotechnology Co., Ltd. (Shanghai, China) and Sigma Chemical Co., Ltd. (St. Louis, MO, USA), respectively.

*Rhei Radix et Rhizoma*, *Coptidis Rhizoma* and *Scutellariae Radix* were combined in a ratio of 2:1:1 and soaked with distilled water (1:10, w/v) for 30 min. Reflux extraction of the mixture was performed three times and the time of extraction were set as 60, 40, and 40 min, respectively. The extracts were consolidated and condensed before being vacuum-dried.

### Preparation of standard and sample solutions

Five reference standards including emodin-8-glucoside (batch number: P29A9F69021, purity > 98%), emodin (batch number: T17O11F127680, purity > 98%), baicalein (batch number: C06M11Y112461, purity > 98%), baicalin (batch number: Z28S11X125952, purity > 98%), and berberine (batch number: S01A10K94340, purity > 98%) were supplied by Shanghai Yuanye Biotechnology Co., Ltd.

For UHPLC-MS analysis, 57.62 mg SHX extraction was weighed and ultrasonicated in 5 mL deionized water for 40 min, subsequently, a 0.22 μm nylon filter was used to filter the resultant dispersion. The standard stock solution with a concentration of 100 g/mL was prepared by dissolving reference standards mentioned above in methanol (chromatographically pure, Fisher Chemical, Fisher, USA) and filtered before detection.

### Analysis of chemical constituents in Sanhuang Xiexin Decoction by UPLC-LTQ-Orbitrap MS

Acetonitrile and methanol of the high-performance liquid chromatography (HPLC) grade were purchased from Fisher Chemical (Fisher, USA), while formic acid of the HPLC grade was purchased from Anaqua Chemicals Supply (Wilmington, DE, USA). The Milli-Q system (Millipore, Bedford, MA, USA) was used to create deionized water.

The analysis was carried out using an LTQ-Orbitrap XL mass spectrometer and an Ultimate 3000 ultra-high performance liquid chromatography system from Thermo Scientific in the USA. A 50 mm × 2.1 mm, 1.7 m ACQUITY UPLC BEH C18 column was used for chromatographic separation. The mobile phase was made up of A (acetonitrile) and B (water with 0.1% formic acid), and the gradient utilized was as follows: 0–1 min, 5% A; 1–8 min, 5%–17% A; 18–25 min, 23–33% A; 30–33 min, 60–90% A; 33–36 min, 90% A; 36–37 min, 90–5% A; 37–40 min, 5% A. The flow rate was 0.3 mL/min, the detection wavelength was 280 nm, the column temperature was 30 ℃, and the injection volume was 2 μL.

The electrospray ionization source on the LTQ-Orbitrap XL mass spectrometer allowed it to run in positive and negative ion modes. The following settings were made for the mass spectrometer analysis’s optimal parameters: m/z 100–1500 scan range; 350 ℃ for the ion source; 350 ℃ for the capillary; 40 ARB for the sheath gas flow; 20 ARB for the auxiliary gas; and 110 (–110) V for the tube lens. Source voltages are 4.0 kV or 3.0 kV for positive ion modes and 25 V or −35 V for negative ion modes, respectively. Thermo Scientific’s Xcalibur 2.1 software was used to evaluate the data.

### UPLC-Q-Extractive-Orbitrap MS analysis of plasma samples with treatment of Sanhuang Xiexin Decoction

Plasma samples from rats that were orally administered Sanhuang Xiexin Decoction extraction were examined using the UPLC-MS technique with the aim of identifying chemicals capable of entering the bloodstream and functioning as active ingredients. After being housed in the room for 1 week, eight male SD (Sprague–Dawley) rats (weighing between 230 and 250 g) were fasted for 12 h with free access to water before the experiment. The rats were randomly divided into two groups (group A, treatment group for dosed rat plasma, n = 4; group B, control group for blank rat plasma, n = 4). Then, 4 rats from group A received the prepared suspension of SHX orally at a dose of 21 g/kg, whereas 4 additional rats from group B received water. At 15, 30, 60, 90, and 120 min after administering SHX, blood samples were taken from the retro-orbital plexus using ethylenediaminetetraacetic acid (EDTA) 1.5 mL polythene tubes. The supernatant was then collected after the tubes were spun at 4,000 rpm for 10 min. Then mix the plasma sample of the same time point of rats in same group.

Plasma (300 μL) was divided into 2 mL tubes, to which acetonitrile was added 3 times, vortexed for 3 min, and then centrifuged at 12,000 rpm for 15 min. The residue was dissolved in 100 μL of acetonitrile and vortexed for 1 min after the supernatant had been evaporated to dryness in the centrifuge concentrator. The supernatant was centrifuged at 12,000 rpm for 15 min, transferred to auto sampler vials, and a 5 μL aliquot was injected into the ultra-high performance liquid chromatography coupled with Q Exactive HF-X Hybrid Quadrupole-Orbitrap mass spectrometer equipped with an electrospray ionization source for analysis (Thermo Scientific, USA).

An ACQUITY UPLC BEH C18 column (100 mm × 2.1 mm, 1.7 μm) (Waters, USA) was used to achieve chromatographic separation. The mobile phase contained the components A (acetonitrile) and B (0.1% formic acid in water, v/v), and the gradient utilized was as follows: 0–1 min, 5%; 1–8 min, 5–17%; 8–18 min, 17–23%; 18–25 min, 23–33%; 30–33 min, 60–90%; 33–36 min, 90%; 36–37 min, 90–5%; 37–42 min, 5%. The flow rate was 0.3 mL/min, the detection wavelength was 280 nm, the column temperature was 30 ℃, and the injection volume was 5 μL.

Positive and negative ions were produced by using the electrospray ionization source of the mass spectrometer. Following is a list of the optimized parameters. Scan range of m/z 100–1200; ion source temperature of 350 ℃; probe heater temperature of 350 ℃; capillary temperature of 320 ℃; sheath gas flow rate of 40 arb; auxiliary gas flow rate of 10 arb. Spray voltage options include 4000 V for positive ion modes and 3500 V for negative ion modes. Xcalibur 2.1 software from Thermo Scientific was used to evaluate the data.

### Animals and experimental protocol

Fifty-six male C57BL/6 J mice that were six weeks old and weighed 20 ± 2 g were acquired from Sibeifu (Beijing) Biotechnology Co., Ltd. (grade SPF, Certificate No: SCXK (Jing) 2019–0010). The mice were kept at 23 ± 2 °C and 60–70% humidity with a 12 h light/dark cycle. The high fat diet had 60% of its calories from fat and containing 5.24 kcal/g, while the regular chow was a standard chow diet comprising 3.65 kcal/g. The regular chow was purchased from SBF (Beijing) Biotechnology Co., Ltd. (Beijing, China, Certificate No: SCXK (Jing) 2019-0010). The high fat diet was purchased from Beijing Huafukang Biotechnology Co., Ltd. (Beijing, China, Certificate No: SCXK (Jing) 2019-0010). Medical and Experimental Animal Ethics Committee of Beijing University of Chinese Medicine accepted the animal protocol for this study (BUCM-4–2021102003-4054).

The Resource Equation Method was used to estimate the sample size of animal experiments. According to the formula, n = 10/k + 1 (k = number of groups, n = number of subjects per group), the minimum number of animals in each group is three mice, and the total sample size is at least 21 mice. In order to obtain sufficient experimental data, a total of 56 mice were used for the experiments and randomly divided into six groups with eight individuals each: control group (n = 8), HFD (n = 8), SHX 2.5 g/kg (n = 8), SHX 5 g/kg (n = 8), SHX 10 g/kg (n = 8), Rosiglitazone 10 mg/kg (n = 8) and CL 316243 1 mg/kg (n = 8). The control group was given regular chow and all other groups were given high fat diet to induce hyperlipidemia for a total of 8 weeks. Mice in SHX 2.5, 5, and 10 g/kg groups and Rosi 10 mg/kg group were taken relative test samples by intragastric administration for consecutive 4 weeks. Mice in control group and model group received distilled water orally. Before dissection, mice in CL 316243 1 mg/kg group were i.p. injected with 1 mg/kg/d of CL 316243 disodium for consecutive 3 days. The food consumption was measured three times a week. The weight gain was measured once a week.

### Cell culture and differentiation

On Corning 6-well cell culture plates (Corning Incorporated-life Sciences, USA), 3T3-L1 preadipocytes (Cell Resource Center, Peking Union Medical College, Beijing, China) were cultivated in a 5% CO_2_ environment at 37 ℃ and maintained in a low glucose Dulbecco’s Modified Eagle’s medium (DMEM, Gibco, C11995500BT) with 10% fetal bovine serum (FBS, Gibco, 2350404RP), 100 U/mL penicillin, and 100 μg/mL streptomycin (NCM Biotech, China). The 2-day postconfluent 3T3-L1 cells (designated as day 0) were incubated with 10% FBS/high glucose DMEM (HG-DMEM), antibodies, 0.5 mM 3-isobutyl-1-methylxanthine (IBMX, Sigma), 1 μM dexamethasone (Sigma), and 5 μg/mL insulin (Biorign, China) for 3 days (days 0–2). Then the cells were incubated for 2 days in 10% FBS/HG-DMEM with insulin (days 3–4), and thereafter incubated in 10% FBS/HG-DMEM that was changed once every 2 days (days 5–8). Cells receiving SHX extraction were given 10% FBS/HG-DMEM and insulin containing a final concentration of 20, 40, 80 μg/mL SHX extraction in PBS that was changed once every two days (days 3–8).

HIB-1B preadipocytes (Qingqi Biotechnology Development Co., Ltd., Shanghai, China) were grown on Corning 6-well cell culture plates (Corning Incorporated-Life Sciences, USA) in a 5% CO_2_ atmosphere at 37 ℃ and maintained in DMEM (HYCLONE, SH30022.01) supplemented with 10% FBS, 100 U/mL penicillin, and 100 μg/mL streptomycin. The 2-day postconfluent HIB-1B cells (designed as day 0) were incubated with 4% FBS/HG-DMEM and antibodies, 0.5 mM IBMX, 250 nM dexamethasone, 10 nM T3 and 170 nM insulin for 2 days (days 0–2). Then the cells were incubated for 6 days in 4% FBS/HG-DMEM with 10 nM T3 and 170 nM insulin that was changed once every 2 days (days 3–8). Cells receiving SHX extraction were given 4% FBS/HG-DMEM and insulin containing a final concentration of 20, 40, 80 μg/mL SHX extraction in PBS that was replaced every two days. (Days 3–8).

### Thermal imaging detection

The hair on the scapula of the back of the mice was removed in advance and the skin temperature were tested the next day. The skin temperature around the shoulder (BAT) was measured by infrared thermography (Fluke TiS50 Thermal Imager, Fluke Corporation, USA) in a quiet state. Then the images were analyzed using a specific software package (Smartview4.3).

### Oral glucose tolerance test

The mice were fasted overnight (12 h) and then orally given with 50% D-glucose (2 g/kg). Blood samples were taken from the tail vein at time points of 0, 30, 60, 90, and 120 min after administration, and blood glucose was determined with the glucometer system Accu-Chek Performa (Roche Diagnostic, India).

### Lee’s index detection

Lee’s index was determined using the mice’s body weight and length (the distance between the tip of their nose and their anus).

### Measurement of adipose tissue mass to body weight

Following the completion of the experimental dosing regimen, the mice underwent a 12-h fasting period with ad libitum access to water. Approximately 1 mL of blood was collected under anesthesia and centrifuged at 3500 rpm for 10 min to separate the serum. After achieving sufficient deep anesthesia, the mice were euthanized by cervical dislocation. Anatomical separation was performed, and inguinal white adipose tissue, epididymal white adipose tissue, perirenal white adipose tissue, and brown adipose tissue were collected and accurately weighed. The ratios of WAT mass to body weight (BW) and BAT mass to body weight were calculated.

### Measurement of total cholesterol and triglyceride levels

According the manufacturer’s instructions, biochemical kits (Jiancheng Bioengineering Institute, Nanjing, China) were used to assess the levels of triglycerides and total cholesterol.

### Histology and immunohistochemistry analysis

BAT and iWAT were fixed with 4% neutral-buffered formalin, dehydrated, embedded in paraffin and sectioned. For histological analysis, the sections were deparaffinized and stained with hematoxylin and eosin (H&E) staining. The expression of UCP1 (1:500, sc-518024, Santa Cruz) in BAT and iWAT of mice was determined by immunohistochemistry method. All images were acquired with the microscope (Olympus, Japan). The expression level of UCP1 in BAT and iWAT of mice was quantified using Image Pro Plus 6.0.

### Quantitative real-time PCR analysis

Total RNA of BAT, iWAT, 3T3-L1 and HIB-1B cells was extracted with TRIzol^®^ reagent (Ambion, USA) according to manufacturer’s instructions. Reverse transcription of total RNA (1 μg) was performed with Revert Aid First Stand cDNA Synthesis Kit (Thermo Scientific, USA). Real-time quantitative PCR (qRT-PCR) was performed with a SYBR Green Master Mix (Novoprotein, China). The PCR reaction was conducted in triplicate for each sample using the Step One Real-Time PCR System (Applied Biosystems, USA). The transcription levels of all genes in each sample were normalized to the level of β-actin. The sequences of primer used in this experiment were shown in Table 1. Total DNA of HIB-1B cell was extracted using DNA Extraction Kit (TIANGEN Biotech CO., LTD.) and quantitative qPCR was performed using mitochondrial DNA and genomic DNA-specific primers [17].

**Table 1:** Primer sequences used for quantitative real-time reverse transcription polymerase chain reaction (qRT-PCR)

Gene name	Forward (5’–3’)	Reverse (5’–3’)
*Adiponectin*	TGCAGGTTGGATGGCAGGCA	CCAGCCCCACACTGAACGCT
*Ap2*	AAATCACCGCAGACGACAG	AAATTTCCATCCAGGCCTCT
*Cd137*	GGTGGACAGCCGAACTGTAA	GCTGCTCCAGTGGTCTTCTT
*CEBPα*	ATCCCAGAGGGACTGGAGTT	AAGTCTTAGCCGGAGGAAGC
*Cidea*	CGGGAATAGCCAGAGTCACC	TGTGCATCGGATGTCGTAGG
*Cpt-1β*	ATGTATCGCCGCAAACTG	CCTGGGATGCGTGTAGTG
*Cyto-c*	CCAAATCTCCACGGTCTGTTC	ATCAGGGTATCCTCTCCCCAG
*FAS*	CAGTATAAGCCCAAGGCCAA	TAGCCCTCCCGTACACTCAC
*Fatp1*	CGCTTTCTGCGTATCGTCTG	GATGCACGGGATCGTGTCT
*Nrf1*	TGTTTGGCGCAGCACCTTT	CGCAGACTCCAGGTCTTCCA
*Nrf2*	GCCTTCCTCTGCTGCCATTA	AACTCCACCGTGCCTTCAGT
*Pgc-1α*	ATGAATGCAGCGGTCTTAGC	AACAATGGCAGGGTTTGTTC
*Pparγ*	TTCAGAAGTGCCTGGCTGTG	TCTTTCCTGTCAAGATCGCC
*Prdm16*	CAGCACGGTGAAGCCATTC	GCGTGCATCCGCTTGTG
*Resistin*	TCATTTCCCCTCCTTTTCCT	GGCTGCTGTCCAGTCTATCC
*Tbx1*	TGAAGAAGAACCCGAAGGTGG	ACTTGGAACGTGGGGAACATT
*Tfam*	GAAGAACGCATGGAGGAGAG	TTCTGGGGAGAGTTGCAGTT
*Tmem26*	AGTGTGAGCAAGAACTCGGG	GATGGCCGGAGAAAGCCATT
*Ucp1*	CGCAGGGAAAGAAACAGCAC	GTTCCAGGATCCAAGTCGCA
*β-actin*	ACTCCTATGTGGGTGACGAGG	CACACGCAGCTCATTGTAGAAG

### Oil red O staining

After being cleaned with phosphate buffered saline (PBS), the cells were fixed with 4% tissue fixative at 37 ℃ for an hour and washed with PBS for 3 times. The cells were added with 1 mL oil red O working solution and stained for 2 h, followed by washing 3–5 times with PBS for 5 min. Then the cells were placed under a microscope to observe the lipid droplet staining.

### Immunofluorescence staining

Cells were cultured on poly-L-lysine-pretreated slides. For mitochondrial staining, 3T3-L1 and HIB-1B cells were treated with growth medium with 200 nM and 100 nM respectively of MitoTracker^®^ Red CMXRos (Cell Signaling Technology, USA) respectively for 30 min at 37 ℃. After incubation, the cells were fixed in 4% paraformaldehyde for 15 min, then washed 3 times with PBS for 5 min. The fixed adipocytes were permeabilized with 0.25% Triton X-100 and blocked with 1% BSA in PBST for 1 h. Then, being exposed to polyclonal anti-UCP1 antibody for an entire night at 4 ℃, adipocytes were subsequently exposed to FITC-conjugated secondary antibody.

### Statistical analyses

All data are expressed as the mean ± standard error of the mean (SEM), and data were analyzed using SPSS 29.0 software or GraphPad Prism 7.03. For n ≥ 5, use the Q-Q plot and Brown-Forsythe test to test the normality and homogeneity of variance of the data, If the data showed normality and equal variation, the significance of the difference. One-way ANOVA or the unpaired, two-tailed Student t-test were used to establish statistical significance; values of *P* < 0.05 were regarded as significant. If the data showed non-normal distribution or biased variation, statistical significance was analyzed by Kruskal Wallis test; values of *P* < 0.05 were regarded as significant.

## Results

### Analysis of chemical constituents in Sanhuang Xiexin Decoction by UPLC-LTQ-Orbitrap MS

**Fig. 1:**
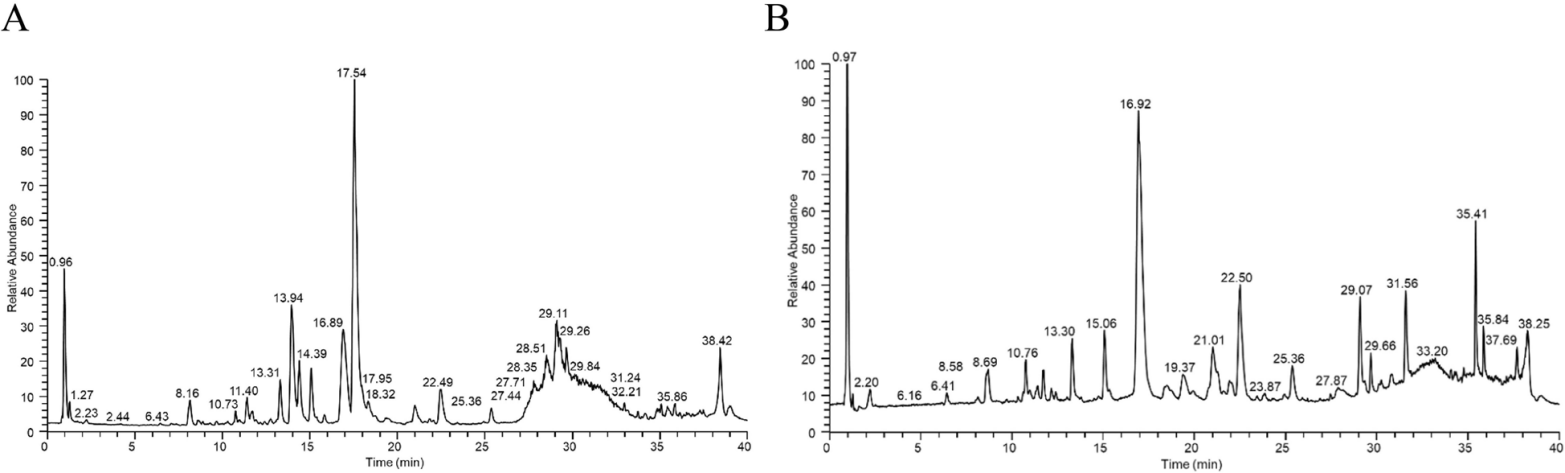
58 compounds were detected in Sanhuang Xiexin Decoction by UPLC-LTQ-Orbitrap MS method. **A** Positive ion mode. **B** Negative ion mode

As a high-resolution mass spectrometry technique, the comprehensive identification of chemicals in complex systems, like traditional Chinese medicine, has been made possible by the extremely sensitive and selective LTQ-Orbitrap-MS technique[18]. Anthraquinones and flavonoids from Rhei rhizome and Scutellariae radix showed good mass spectral response in negative ion mode, while alkaloids from Scutellariae radix showed more fragments in positive ion mode (Fig. 1). Utilizing elemental composition analysis, MS/MS data, literature, and chromatographic retention durations with a 5 ppm error margin, we identified a total of 58 compounds in SHX, listed in Additional file 1: Supplementary Table 1, which include 10 anthraquinones (e.g. emodin-8-O-β-D-glucoside, emodin, rhein, physcion, chrysophanol, and etc.), 3 tannins ingredients (e.g. gallic acid, epicatechin, and etc.), 16 alkaloids (e.g. berberine, magnoflorine, coptisine, jatrorrhizine, palmatine, and etc.), 24 flavonoids and their glycosides (e.g. baicalin, baicalein, apigenin, oroxylin A 7-O-β-D-glucuronide, wogonin, and etc.) and 5 other compounds derived from Coptidis Rhizoma. By identifying the chemical constituents of SHX, the compounds that serve as the pharmacodynamic material basis are clarified preliminarily.

### UPLC-Q-Extractive-Orbitrap MS analysis of plasma samples with treatment of Sanhuang Xiexin Decoction

**Fig. 2:**
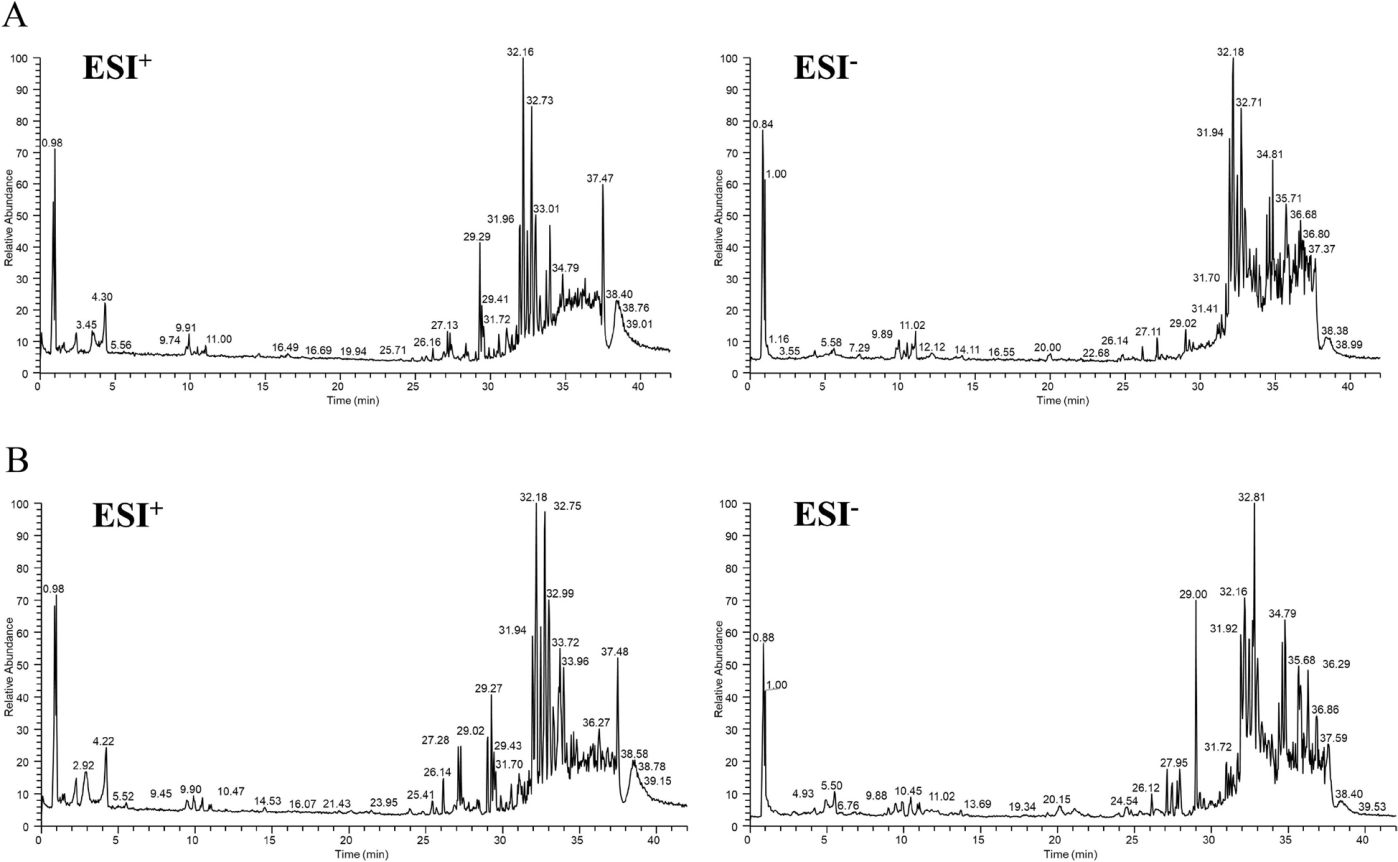
31 prototype compositions and 67 metabolites were identified in rat plasma using UPLC-Q-Extractive-Orbitrap MS analysis. **A** Rat plasma blank. **B** Rat plasma sample

To investigate the active constituents responsible for the efficacy of the SHX extraction, rat plasma samples that had been administered orally were examined using a UPLC-MS^n^ technique. The goal was to find chemicals that could enter the bloodstream and function as active ingredients. A total of 98 compounds, comprising 31 prototype compositions and 67 metabolites, were identified by fundamental composition analysis within a 5-ppm error range by comparing the retention duration, accurate ions, and fragment ions of SHX extraction-containing plasma to blank plasma. Among them, 39 components including prototype compositions and metabolites identified from Rhei radix et rhizoma, most of the metabolites were generated by carboxylation, hydroxylation, methylation, glucuronidation and sulfate esterification of emodin and rhein. 37 components were identified from Scutellariae radix, the main components of which were flavonoids and their metabolites. 22 components were identified from Coptidis rhizome, with alkaloids and their metabolites as the main active components. The following figure depict the total ion chromatograms (TICs) of rat plasma samples taken after oral administration of SHX extraction and a blank rat plasma sample(Fig. 2A, B). In Additional file 1: Supplementary Table 2, the mass spectrometry data for 98 components are compiled.

### Sanhuang Xiexin Decoction decreased body weight and fat mass in obese mice caused by the high fat diet without changing food consumption

**Fig. 3:**
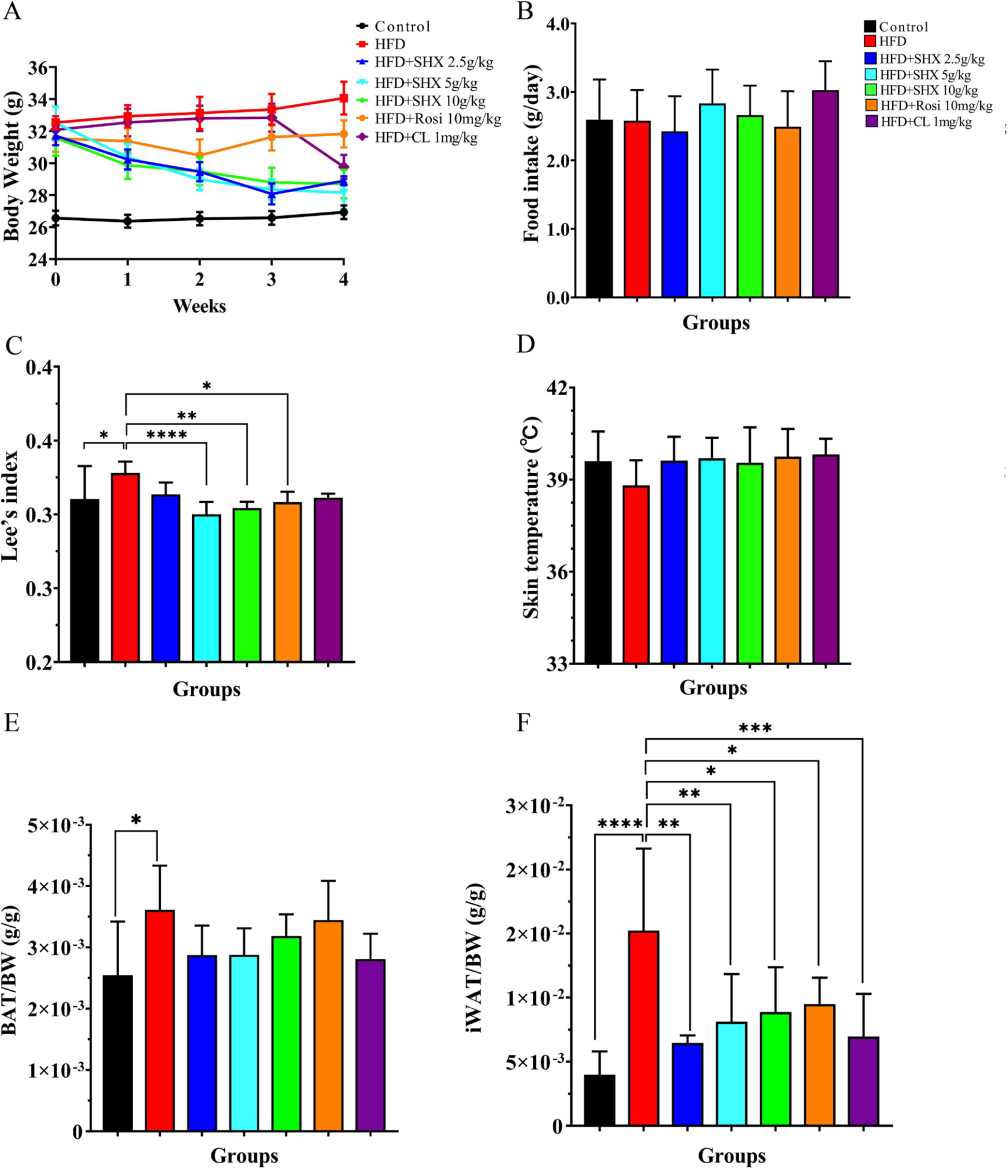
Sanhuang Xiexin Decoction orally administered at doses of 2.5, 5, and 10 g/kg could reduce body weight and fat mass in obesity mice inducing by high fat diet without changing food intake. **A** Body weight. **B** Food intake. **C** Lee’s index. **D** Interscapular BAT surface temperature. **E** The ratio of BAT/BW. **F** The ratio of iWAT/BW. SHX, Sanhuang Xiexin Decoction; BAT, brown adipose tissue; iWAT, inguinal white adipose tissue. Data are expressed as mean ± SE, with n = 5 ~ 8. ^*^P < 0.05, ^**^P < 0.01, ^***^P < 0.001, ^****^P < 0.0001 *vs*. HFD group

In comparison with the control mice, HFD treated obese model mice showed a significantly increased in body weight, Lee’s index, and ratios of iWAT/BW, and BAT/BW. However, treatment with SHX at doses of 2.5, 5 and 10 g/kg led to a significant loss in body weight at week 2 (by 11%, 18%, and 11%, *P* = 0.030, *P* = 0.009, *P* = 0.031, respectively), week 3 (by 14%, 23%, and 15%, *P* = 0.001, *P* = 0.0007, and P = 0.0026, respectively), and week 4 (by 15%, 17%, and 16%, *P* = 0.0022, *P* = 0.00009, and* P* = 0.0004, respectively) (Fig. 3A). SHX treatment at doses of 2.5, 5, and 10 g/kg did not change the food intake in obesity mice (Fig. 3B).

Lee’s index applied to assess the level of obesity in adult obese mice was significantly decreased by 8.452% and 7.78% with SHX treatment at doses of 5 and 10 g/kg, respectively (*P* = 0.000027 and *P* = 0.0033) (Fig. 3C).

The thermogenic effects of non-shivering are mediated by brown adipose tissue, which also plays a role in absorbing glucose and lipids and boosting energy expenditure. In this study, the body temperature of interscapular BAT surface was measured by infrared thermography. Treatment with SHX at 5 g/kg and 10 g/kg resulted in mild increase skin temperature compared to HFD treated mice (Fig. 3D). However, when compared to obese mice, SHX did not change the ratio of BAT/BW (Fig. 3E).

Compared with HFD-treated mice, treatment with SHX at doses of 2.5, 5, and 10 g/kg significantly reduced ratios of iWAT/BW by 58%, 46% and 42%, respectively (*P* = 0.001839, *P* = 0.004933, *P* = 0.015920) (Fig. 3F).

### Sanhuang Xiexin Decoction ameliorated abnormal blood lipid and glucose in obese mice caused by the high fat diet

**Fig. 4:**
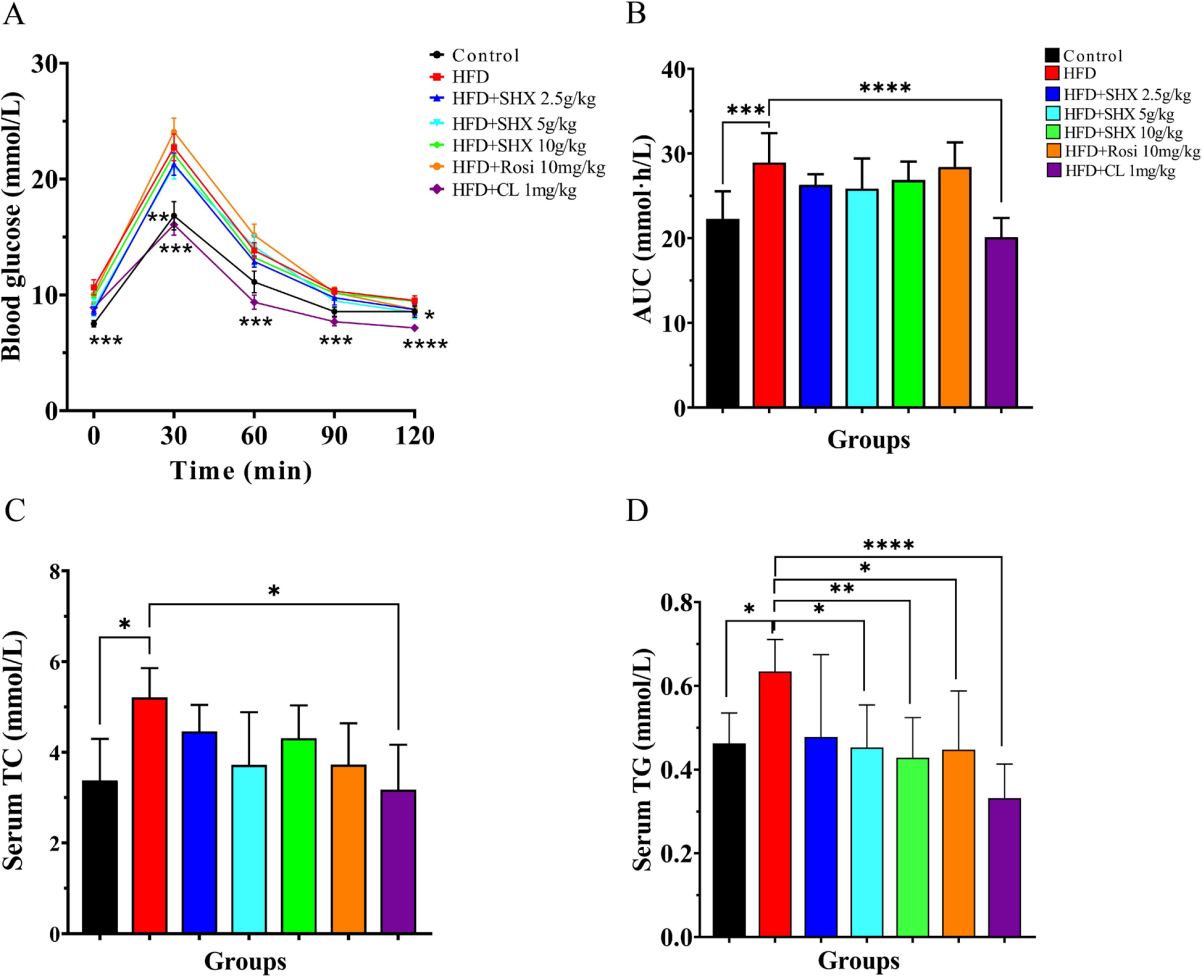
Sanhuang Xiexin Decoction could increase glucose tolerance and decrease blood TC and TG levels in obese mice. **A** Oral glucose tolerance test (OGTT). With 4-week SHX treatment, the mice were administrated with 2 g/kg glucose after 12-h fasting, blood samples were taken at time points of 0, 30, 60, 90, and 120 min after oral gavage. **B** Quantification of AUC from OGTT. **C** Serum TC concentration. **D** Serum TG concentration. Data are expressed as mean ± SE, with n = 5 ~ 8. ^*^P < 0.05, ^**^P < 0.01, ^***^P < 0.001, ^****^P < 0.0001 *vs*. HFD group

In this research, we looked into whether SHX enhanced the ability of obese mice to tolerate glucose. In comparison with the control group, blood glucose and the AUC (area under the curve) index in model group considerably increased (*P* = 0.00019). SHX at 5 g/kg significantly reduced blood glucose levels (*P* = 0.0167) at time point 120 min, when compared to HFD mice. However, the AUC index did not show a remarkable change in SHX-treated groups (Fig. 4A, B).

We also examined whether SHX improved blood lipids in obese mice. A persistent high fat diet may cause metabolic lipid disorders and ectopic lipid deposition. Compared to the control group, serum TC (total cholesterol) and TG (triglyceride) contents of HFD fed mice significantly increased by 35% and 27% (*P* = 0.049, *P* = 0.023), respectively. When compared with HFD-treated mice, SHX at 5 g/kg remarkably decreased serum TG by 28% (*P* = 0.029), and SHX at 10 g/kg significantly decreased serum TG level by 32% (*P* = 0.008) (Fig. 4C, D).

### Sanhuang Xiexin Decoction induced browning of iWAT in obese mice caused by the high fat diet

**Fig. 5:**
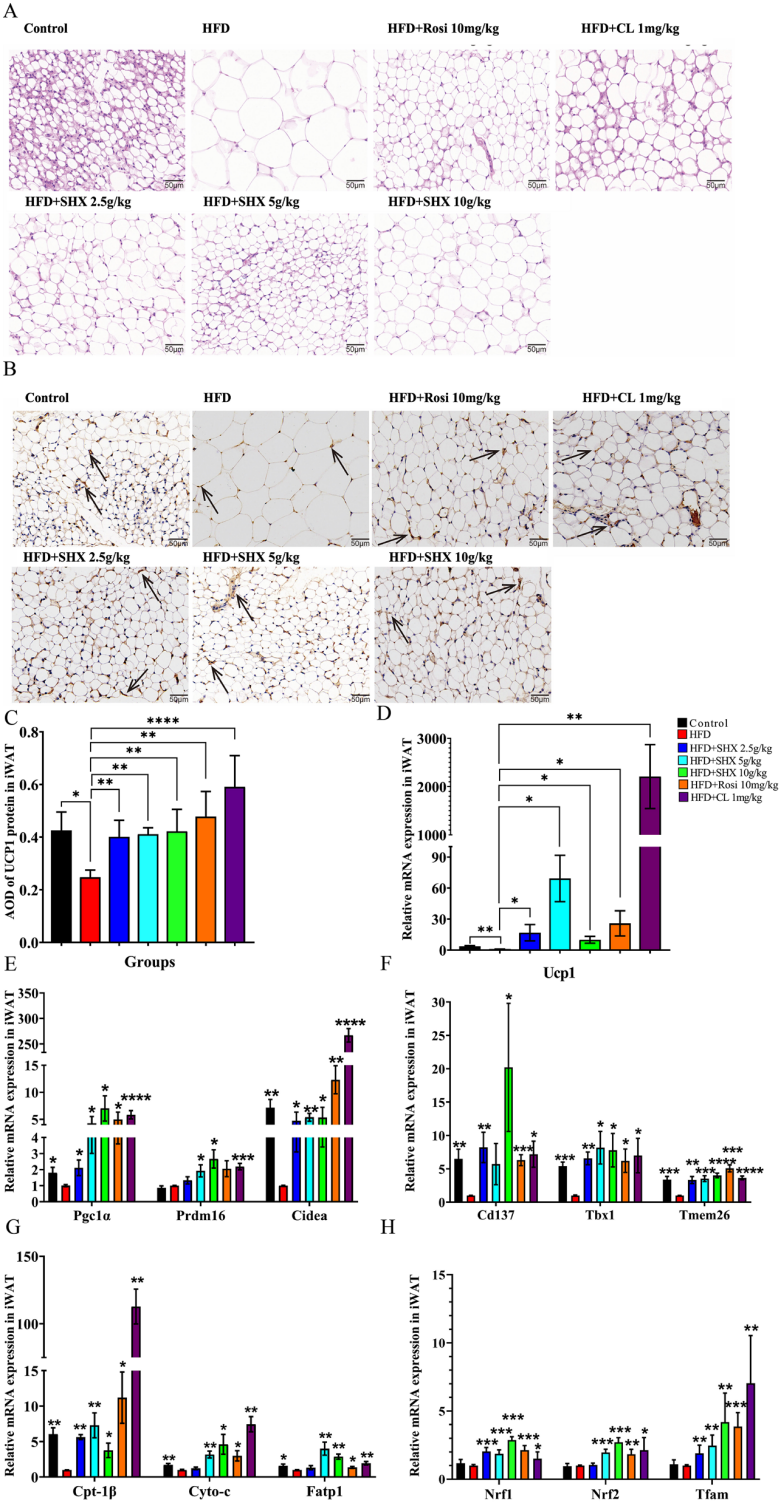
Sanhuang Xiexin Decoction induced browning of iWAT in mice fed with high fat diet. **A** H&E staining of iWAT. Images were observed under a light microscope at 400 × magnification (Scale bar = 50 μm). **B** Immunohistochemistry staining of UCP1 of iWAT. **C** Relative expression of UCP1 protein in iWAT. **D** The relative mRNA expression level of *Ucp1* mRNA in iWAT. **E** Relative expression of genes related to thermogenesis including *Pgc1α*, *Prdm16*, and *Cidea* in iWAT. **F** The gene expression of beige adipocytes markers including *Cd137*, *Tbx1*, *Tmem26* in iWAT. **G** The gene expression related to fatty acid oxidation including *Cpt-1β*, *Cyto-c*, and *Fatp1* in iWAT. **H** The gene expression of mitochondrial biogenic transcription factors including *Nrf1*, *Nrf2*, and *Tfam* in iWAT. Data are expressed as mean ± SE, with n = 4 ~ 6. ^*^P < 0.05, ^**^P < 0.01, ^***^P < 0.001, ^****^P < 0.0001 *vs*. HFD group

In this study, we examined the expression of the thermogenic protein UCP1 and a number of beige adipocyte markers, as well as the shape of iWAT. In comparison with control group, the width of fat cells rose and the density of cells per unit area dropped in HFD-induced obese mice. However, in the SHX (2.5, 5, and 10 g/kg) treated groups, the adipocytes of the mice were tiny, compactly organized, with obvious nuclei (Fig. 5A). WAT can differentiate into beige adipocytes in reaction to a number of factors including exercise, cold exposure, etc. Browning of WAT is the term for the phenomena wherein beige adipocytes morphologically and functionally resemble brown adipocytes in many ways. UCP1 expression levels in brown or beige adipocytes are a good indicator of thermogenic potential. We first looked at UCP1 protein expression in iWAT to see whether browning of iWAT was induced following SHX therapy. The protein expression of UCP1 in iWAT of SHX 2.5, 5, and 10 g/kg groups was obviously increased (*P* = 0.005, *P* = 0.0039, and *P* = 0.00199), when compared with HFD group (Fig. 5B, C). In addition, gene expression of *Ucp1* in iWAT of SHX (2.5, 5, and 10 g/kg) treated groups was also increased significantly (Fig. 5D).

Meanwhile, the expression of several genes related to thermogenesis (e.g. peroxisome proliferator activated receptor γ coactivator-1α, positive regulatory domain-containing 16, cell death-inducing DFFA-like effector A) were significantly up-regulated after SHX (2.5, 5, and 10 g/kg) treatment (*Pgc1α*, *P* = 0.046, *P* = 0.026, *P* = 0.028; *Prdm16*, *P* = 0.176, *P* = 0.035, *P* = 0.015; *Cidea*, *P* = 0.044, *P* = 0.001, *P* = 0.047), compared with obese mice (Fig. 5E). As expected, after SHX treatment, the gene expression of beige markers (e.g. cluster of differentiation 137, gene encoding T-box protein 1, transmembrane protein 26) and fatty acid oxidation (e.g. carnitine palmitoyl transferase 1β, cytochrome c, *Fatp1*) was also dramatically up-regulated in iWAT, when compared with the HFD group (*Cd137*, *P* = 0.0099, *P* = 0.158, and *P* = 0.035; *Tbx1*, *P* = 0.0016, *P* = 0.015, *P* = 0.022; *Tmem26*, *P* = 0.001, *P* = 0.00019, *P* = 0.00001; *Cpt-1β*, *P* = 0.0012, *P* = 0.005, *P* = 0.023, *Cyto-c*, *P* = 0.278, *P* = 0.0012, *P* = 0.027, *Fatp1*, *P* = 0.287, *P* = 0.008, and *P* = 0.0042) (Fig. 5F, G).

Similar with brown adipocytes, beige cells densely packed mitochondria. We also investigated mitochondrial biogenesis in iWAT. SHX (2.5, 5, and 10 g/kg) significantly upregulated the manifestation of mitochondrial biogenic transcription factors, such as nuclear respiratory factor 1/2 (NRF1/2) (*Nrf1*, *P* = 0.0001, *P* = 0.0004, and *P* = 0.0005, *Nrf2*, *P* = 0.434, *P* = 0.00022, *P* = 0.00027), and mitochondrial transcription factor A (*Tfam*) (*P* = 0.005, *P* = 0.001, *P* = 0.004), when compared with obese mice (Fig. 5H).

### Sanhuang Xiexin Decoction activated brown adipose tissue in obese mice caused by the high fat diet

**Fig. 6:**
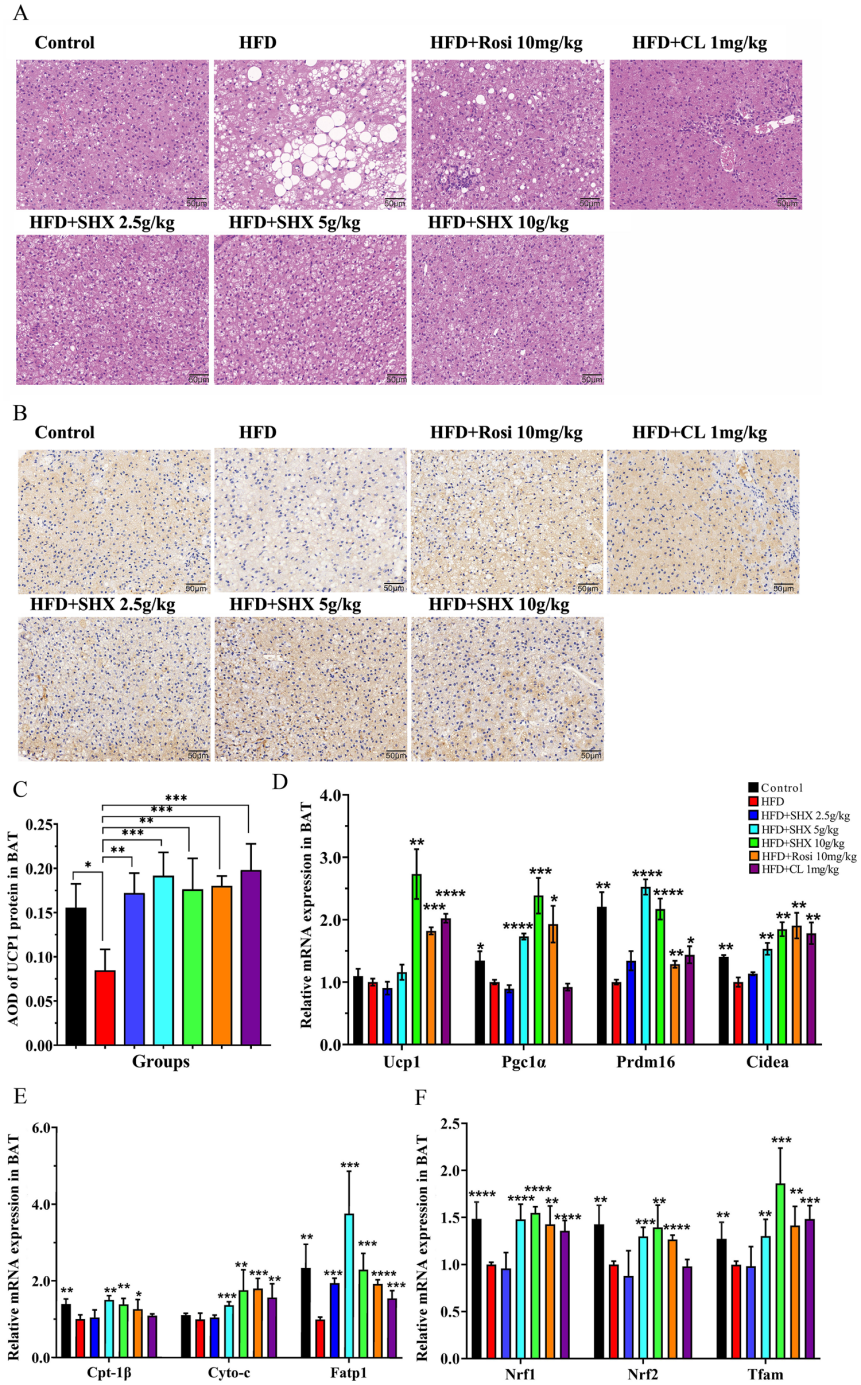
Sanhuang Xiexin Decoction activated brown adipose tissue in mice fed with high fat diet. **A** H&E staining of BAT. Images were observed under a light microscope at 400 × magnification (Scale bar = 50 μm). **B** Immunohistochemistry staining of UCP1 in the BAT. **C** Relative expression of UCP1 protein in BAT. **D** Relative gene expression of BAT-specific markers (*Ucp1*, *Pgc1α*, *Prdm16*, and *Cidea*) in BAT. **E** Relative gene expression of fatty acid oxidation factors (*Cpt-1β*, *Cyto-c*, and *Fatp1*) in BAT. **F** Relative gene expression of mitochondrial biogenesis markers (*Nrf1*, *Nrf2*, and *Tfam*) in BAT. Data are expressed as mean ± SE, with n = 6. ^*^P < 0.05, ^**^P < 0.01, ^***^P < 0.001, ^****^P < 0.0001 *vs*. HFD group

We also looked into BAT’s morphology. The width of fat cells dramatically increased in HFD mice when compared to normal mice, indicating a trend toward BAT whitening. When compared with HFD treated mice, the brown adipocytes of the mice in SHX (2.5, 5, and 10 g/kg) treatment groups were small and tightly arranged, and the number of white adipocytes was reduced (Fig. 6A). The protein expression of UCP1 in BAT of SHX (2.5, 5, and 10 g/kg) groups was remarkably increased (*P* = 0.0025, *P* = 0.00063, and *P* = 0.0026), when compared with HFD group (Fig. 6B, C).

Given that one of the primary methods to boost energy expenditure depends on brown adipocyte stimulation, we investigated whether SHX could activate BAT in vivo. When compared with HFD induced obese mice, SHX 10 g/kg could significantly increase *Ucp1* mRNA expression (*P* = 0.0016), SHX at doses of 5 and 10 g/kg could significantly upregulated *Pgc1α*, *Prdm16*, and *Cidea* (*Pgc1α*,* P* = 0.00000029, *P* = 0.00069; *Prdm16*, *P* = 0.00000031, *P* = 0.00005; *Cidea*, *P* = 0.0013, and *P* = 0.0091) mRNA genes expression (Fig. 6D). Additionally, mice treated with SHX (2.5, 5, or 10 g/kg) showed significantly higher expression of mitochondrial biogenic transcription factors including *Nrf1*, *Nrf2*, Tfam (*Nrf1*, *P* = 0.55409, *P* = 0.000033, *P* = 0.0000039; *Nrf2*, *P* = 0.300, *P* = 0.0004, *P* = 0.00237; *Tfam*, *P* = 0.856, *P* = 0.0023, *P* = 0.00023), as well as fatty acid oxidation genes like *Cpt-1β*, *Cyto-c*, and *Ftap1* (*Cpt-1β*, *P* = 0.643,* P* = 0.0022, *P* = 0.0055; *Cyto-c*, *P* = 0.520, *P* = 0.00069, *P* = 0.0076; *Fatp1*, *P* = 0.00017, *P* = 0.00012, *P* = 0.0002) (Fig. 6E, F).

### Sanhuang Xiexin Decoction induced browning of 3T3-L1 white adipocytes in vitro

The browning of WAT contributes to energy consumption. The expression of brown fat markers was assessed when 3T3-L1 white adipocytes were treated with various concentrations of SHX (20–80 g/mL), in order to study the potential browning effect of SHX. The results revealed that SHX 20–80 μg/mL treatment significantly increased mRNA expressions of *Ucp1*(*P* = 0.177, *P* = 0.042, *P* = 0.013), *Pgc1α* (*P* = 0.005, *P* = 0.001, *P* = 0.568) and *Prdm16* (*P* = 0.046, *P* = 0.051, *P* = 0.967) in 3T3-L1 adipocytes and significantly upregulated the expression of mitochondrial biogenic transcription factors, including *Nrf1*(*P* = 0.054, *P* = 0.011,*P* = 0.681) and *Tfam* (*P* = 0.003, *P* = 0.001, *P* = 0.042). These findings suggested that SHX therapy might encourage white adipocyte browning and thermogenic capacity.

**Fig. 7:**
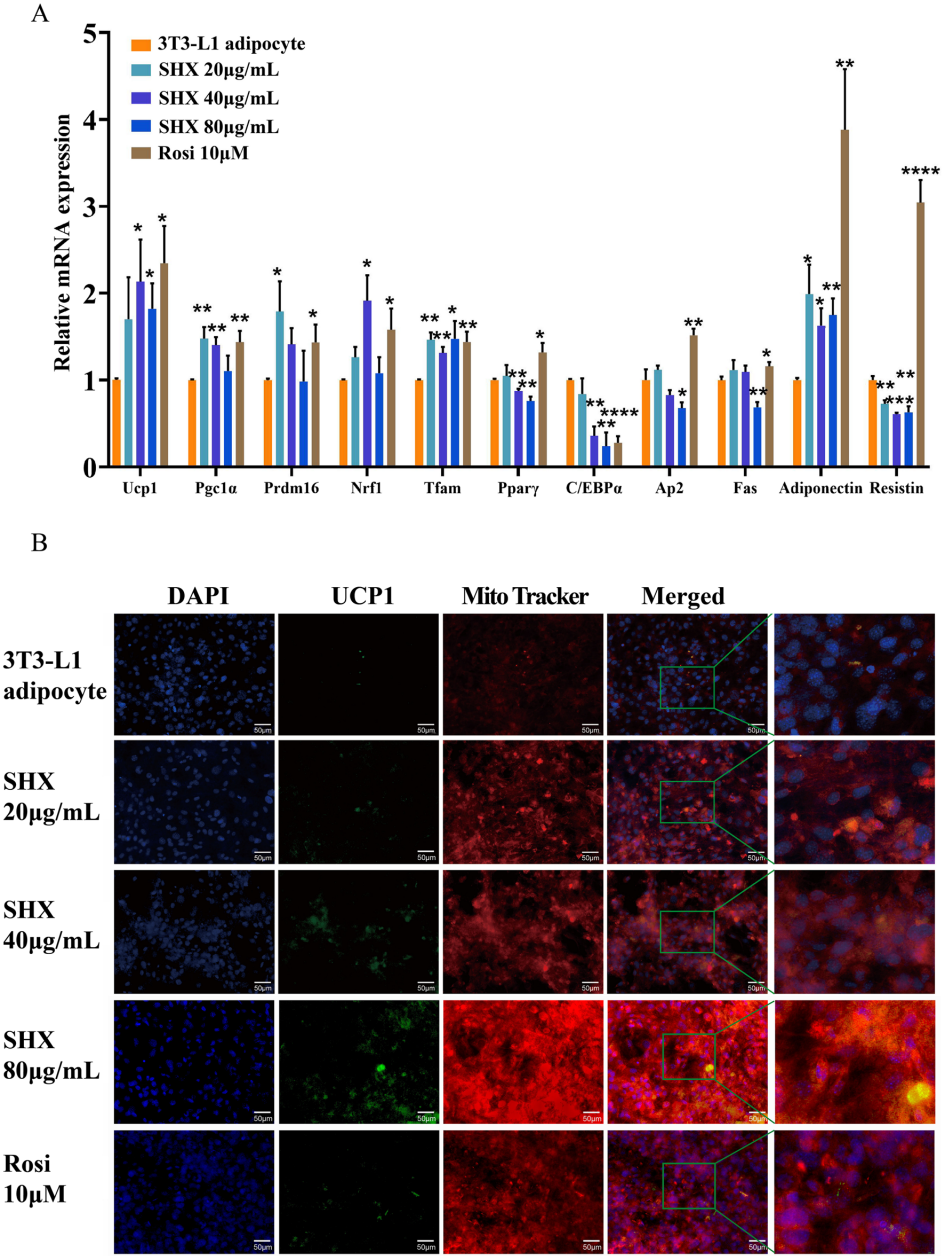
Sanhuang Xiexin Decoction induced browning of 3T3-L1 white adipocytes in vitro. 3T3-L1 white adipocytes were incubated with SHX at concentrations of 20, 40, 80 μg/mL, Rosi 10 μM for 6–7 days. **A** Gene expression related to thermogenesis, adipocyte differentiation and adipokines. **B** Immunofluorescence image of 3T3-L1 adipocytes (400 × magnification). Data are expressed as mean ± SE, with n = 6. ^*^P < 0.05, ^**^P < 0.01, ^***^P < 0.001, ^****^P < 0.0001, *vs.* 3T3-L1 adipocyte group

Adipocyte hypertrophy and hyperplasia were indicators of obesity. Obesity therapy strategies may be able to target the inhibition of adipose differentiation. We examined the impact of SHX on adipogenic markers of 3T3-L1 cells. The mRNA level of *Pparγ* (40 and 80 μg/mL, *P* = 0.001 and *P* = 0.007), CCAAT/enhancer-binding protein/encoding gene α (*C/EBPα*) (40 and 80 μg/mL, *P* = 0.0016, *P* = 0.0074), *Ap2*(80 μg/mL* P* = 0.044) and fatty acid synthase (*Fas*) (80 μg/mL*, P* = 0.002) was significantly decreased after treatment of SHX 20–80 μg/mL, which indicated that SHX could inhibit the differentiation of white adipocyte. According to our findings, different concentrations of SHX treatment considerably boosted the mRNA expression level of adiponectin (*P* = 0.015, *P* = 0.0111, *P* = 0.0028) while dramatically decreasing the mRNA expression level of Resistin (*P* = 0.001, *P* = 0.00013, *P* = 0.0013) (Fig. 7A).

Adipocytes treated with SHX had noticeably more mitochondria, which was demonstrated by improved red fluorescent dye binding to the mitochondria. Meanwhile, the expression of UCP1 was increased in the 3T3-L1 adipocytes(Fig. 7B).

### Sanhuang Xiexin Decoction activated HIB-1B brown adipocytes in vitro

**Fig. 8:**
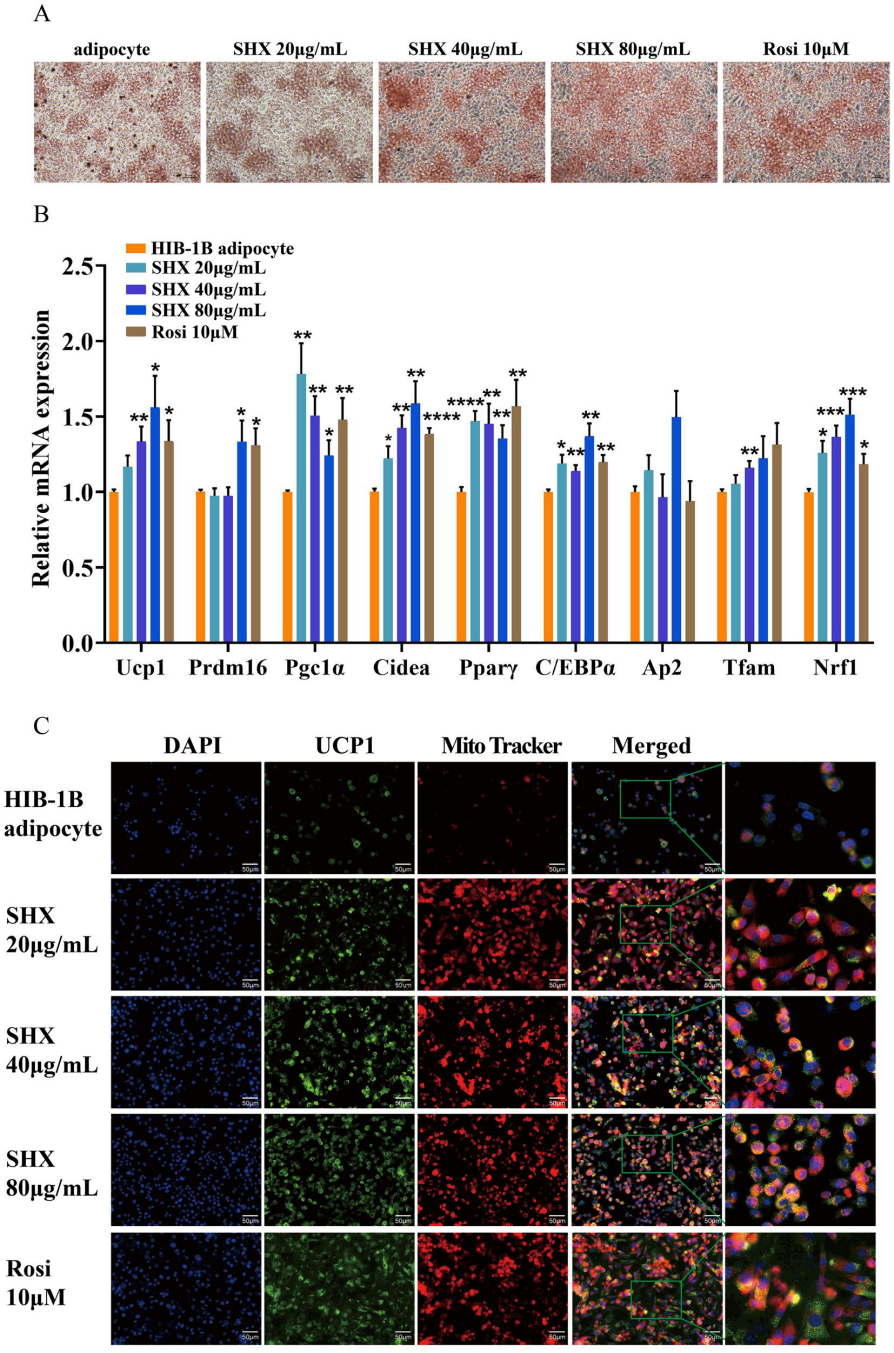
Sanhuang Xiexin Decoction activated HIB-1B brown adipocytes in vitro. HIB-1B brown adipocytes were incubated with SHX at concentrations of 20, 40, 80 μg/mL and Rosi 10 μM for 6–7 days. **A** Representative images of Oil Red O staining of HIB-1B brown adipocytes were taken at 200 × magnification (scale bars = 25 μm). **B** qRT-PCR analysis of genes related to thermogenesis, adipocyte differentiation and mitochondrial biogenesis. **C** Immunofluorescence image of HIB-1B adipocytes (400 × magnification). Data are expressed as mean ± SE, with n = 6. ^*^P < 0.05, ^**^P < 0.01, ^***^P < 0.001, ^****^P < 0.0001, *vs.* 3T3-L1 adipocyte group

We further investigated whether SHX activated HIB-1B brown adipocytes in vitro.Our results demonstrate that SHX at concentration of 20–80 μg/mL could enhance intensity of Oil Red O staining (Fig. 8A). Meanwhile SHX 20–80 μg/mL significantly activated HIB-1B brown adipocytes differentiation and enhanced expression of brown fat-specific marker genes including *Ucp1*(*P* = 0.0508, *P* = 0.0075, *P* = 0.0234), *Prdm16* (*P* = 0.6036, *P* = 0.6183, *P* = 0.0396), *Pgc1α* (*P* = 0.0032, *P* = 0.0028, *P* = 0.0359), and *Cidea* (*P* = 0.023, *P* = 0.0057, *P* = 0.0028), and the expression levels of key adipogenic markers including *Pparγ* (*P* = 0.000092, *P* = 0.0081, *P* = 0.0037), C/EBPα (*P* = 0.0115, *P* = 0.0067, *P* = 0.0015) and Ap2 (*P* = 0.204, *P* = 0.822, *P* = 0.0987) in HIB-1B brown adipocytes. Then we verified levels of mitochondrial biogenesis genes. The expression of Tfam (*P* = 0.404, *P* = 0.0079, *P* = 0.161) and Nrf1 (*P* = 0.011, *P* = 0.00078 and *P* = 0.00068) were elevated with SHX 20–80 μg/mL treatment (Fig. 8B).

Furthermore, SHX (20–80 μg/ml) remarkably enhanced UCP1 expression and mitochondrial expression in HIB-1B brown adipocytes. Based on the above data, SHX activates brown adipocytes by regulating mitochondrial function and UCP1 expression (Fig. 8C).

## Discussion

Sanhuang Xiexin Decoction has been used for over a thousand years in the treatment of hematemesis and epistaxis, including conditions such as gastric hemorrhage and nasal hemorrhage. Three herbal components in this formula are all bitter and cold in nature, exhibiting remarkable heat-clearing effects. Recently, multiple studies are focusing on the lipid-lowering effects of Sanhuang Xiexin decoction. However, the complex composition of this herbal formula and biological mechanisms have not yet been fully elucidated.

Our study first identified the key chemical components responsible for the lipid-lowering effects of Sanhuang Xiexin Decoction. Totally, 31 prototype compositions and 67 metabolites were identified, most of which were flavonoids, alkaloids and their metabolites, including Emodin, Emodin-8-β-D-glucoside, Rhein, Berberine, Baicalin, Baicalein, etc.

Excessive amounts of visceral adipose tissue and of ectopic fat largely define the cardiovascular disease risk of overweight and moderate obesity [19]. SHX could remarkably decrease the body weight of obesity mice, and inhibit ectopic fat accumulation, including decreasing indexes of iWAT/BW, eWAT/BW and pWAT/BW, as well as reducing the size of lipid droplets in iWAT. However, the anti-obesity effect of SHX was not achieved by reducing food intake in obesity mice.

Adipose tissue, once regarded as an insignificant tissue mass, is now recognized as a critical metabolic organ that regulates whole-body energy homeostasis via the regulation of energy storage and dissipation and secretion of metabolically-active factors, and is closely linked to the underlying obesity, diabetes, and related metabolic disorders [20]. Extensive evidence indicates that in rodents, BAT thermogenesis is essential for chronic adaptation to cold environments, and its activation leads to resistance to obesity and protects against numerous other negative metabolic consequences [21]. And the browning of WAT is a process that converts energy-storing white adipocytes into beige adipocytes with the capacity to produce heat, which can lead to increased energy expenditure and represents an attractive strategy for treating obesity.

In vivo experiment, we discovered that SHX treatment induced the browning of iWAT in obese mice, which was reflected in increased expression of brown fat specific genes and beige specific markers. In addition, SHX treatment significantly upregulated the expression of genes related to fatty acid oxidation and mitochondrial biogenesis. The above results clearly indicated that SHX could promote browning of WAT, resulting in increased energy consumption in obese mice. Notably, SHX could also promote thermogenesis and energy consumption through activation of BAT.

In vitro experiment, HIB-1B brown adipocytes and 3T3-L1 white adipocytes were cultured, SHX extraction could directly target adipocytes. SHX administration could inhibit adipogenesis in 3T3-L1 adipocytes by down-regulating adipogenic transcription factors, while it could promote the differentiation of HIB-1B preadipocyte. Meanwhile, SHX extraction could regulated several adipokines, including adiponectin, resistin, and leptin, which related its efficacy on lipid metabolism and insulin sensitivity.

## Conclusion

Our study suggested that Sanhuang Xiexin Decoction can ameliorate metabolic abnormalities associated with obesity using both in vivo and in vitro experiments. And the potential mechanism was related to inducing browning of white adipose tissue and activating brown adipose tissue. The resulted indicated that Sanhuang Xiexin Decoction could be used as a potential therapeutic candidate for the treatment of obesity and its complications.

## Supplementary Information

Additional file 1: Supplementary Tables 1 and 2

## Data Availability

Not applicable.

## Abbreviations

SHX: Sanhuang Xiexin Decoction
WHO: World Health Organization
WAT: White adipose tissue
iWAT: Inguinal white adipose tissue
eWAT: Epididymal white adipose tissue
pWAT: Perirenal white adipose tissue
BAT: Brown adipose tissue
UCP1: Uncoupling protein 1
HPLC: High-performance liquid chromatography
SD: Sprague–Dawley
EDTA: Ethylenediaminetetraacetic acid
DMEM: Dulbecco’s Modified Eagle’s medium
FBS: Fetal bovine serum
HG-DMEM: High glucose DMEM
IBMX: 3-Isobutyl-1-methylxanthine
BW: Body weight
qRT-PCR: Real-time quantitative PCR
H&E: Hematoxylin and eosin
PBS: Phosphate buffered saline
SEM: Standard error of the mean
TICs: Total ion chromatograms
HFD: High fat diet
AUC: Area under the curve
TC: Total cholesterol
TG: Triglyceride
OGTT: Oral glucose tolerance test
*Tfam*: Transcription factor A
*NRF1*: Nuclear respiratory factor 1
*NRF2*: Nuclear respiratory factor 2
*Fas*: Fatty acid synthase
*C/EBPα*: CCAAT/enhancer-binding protein/encoding gene α
*Tmem26*: Transmembrane protein 26
FDG: F-fluorodeoxyglucose
CT: X-ray computed tomography
PET: Positron emission tomography
*Cd137*: Cluster of differentiation 137
*Prdm16*: PR domain containing 16
*Pparγ*: Peroxisome proliferator-activated receptor γ
*Pgc-1α*: Peroxisome proliferator-activated receptor γ coactivator 1-alpha
*Cpt-1β*: Carnitine palmitoyl transferase 1β
*Cyto-c*: Cytochrome c
*Tbx1*: Gene encoding T-box protein 1
