# Supplementary material for: Sanhuang Xiexin Decoction ameliorates lipid disorders in obese mice via inducing browning of white adipose tissue and activating brown adipose tissue
Source: Chin Med. 2025 May 9;20:60. doi: 10.1186/s13020-025-01111-3 (PMC12065319; doi:10.1186/s13020-025-01111-3)
Supplement: Supplementary file 1 — Additional file 1: Supplementary Tables 1 and 2 [file 13020_2025_1111_MOESM1_ESM.pdf]

SUPPLEMENT TABLE 1 Chemical constituents identified in Sanhuang Xiexin Decoction.

NO.	t_R/min	Identification	Compound formula	Proposal ions	Experimental m/z	Theoretical m/z	Erro ppm	MS^2 data	Source
1*	21.92	Emodin-8-β-D-glucoside	$C_{21}H_{20}O_{10}$	$[M-H]^-$	431.09802	431.0973	1.73	269.05396, 241.16629, 225.12933, 181.05165	A
2*	31.56	Emodin	$C_{15}H_{10}O_5$	$[M-H]^-$	269.04523	269.0444	2.90	241.01561, 224.99785, 197.06908, 180.99837	A
3	27.81	Aloe-emodin	$C_{15}H_{10}O_5$	$[M-H]^-$	269.04520	269.0444	2.79	241.11205, 225.13585, 183.00221	A
4	29.03	Rhein	$C_{15}H_8O_6$	$[M-H]^-$	283.02490	283.0237	4.19	257.05954, 238.93741, 210.99399, 183.01797	A
5	21.30	Aloe-emodin-3-hydroxymethyl-O-β-D-glucopyranoside	$C_{21}H_{20}O_{10}$	$[M-H]^-$	431.09808	431.0973	1.87	311.18512, 269.13803, 268.02744	A
6	21.81	Emodin-1-O-glucoside	$C_{21}H_{20}O_{10}$	$[M-H]^-$	431.09802	431.0973	1.73	269.04037, 240.08905	A
7	20.98	Chrysophanol-8-O-glucoside	$C_{21}H_{20}O_9$	$[M-H]^-$	415.10269	415.1024	1.23	277.05981, 253.17551	A
8	29.07	Physcion	$C_{16}H_{12}O_5$	$[M-H]^-$	283.06010	283.0601	0.92	268.02350, 238.93741, 223.11478	A
9	29.30	Chrysophanol	$C_{15}H_{10}O_4$	$[M-H]^-$	253.05043	253.0495	3.54	225.07896, 210.19923, 181.04597, 153.09137	A
10	11.68	2-(2′-hydroxypropyl)-5-methyl-7-hydroxychromone	$C_{13}H_{14}O_4$	$[M-H]^-$	233.08148	233.0808	2.77	188.94719	A
11	2.21	Gallic acid	$C_7H_6O_5$	$[M-H]^-$	169.01398	169.0131	4.91	124.82675	A
12	4.78	Epicatechin	$C_{15}H_{14}O_6$	$[M-H]^-$	289.07138	289.0707	2.48	271.10870, 245.14032, 205.09503, 203.11301	A
13	1.38	6-O-galloylglucose	$C_{13}H_{16}O_{10}$	$[M-H]^-$	331.06647	331.0660	1.50	211.00101, 169.01398	A

14*	17.54	Berberine	$C_{20}H_{18}NO_4$	$[M]^+$	336.12341	336.1230	1.12	320.13086, 306.19934, 292.14346, 278.07513	B
15	8.16	Magnoflorine	$C_{20}H_{24}NO_4$	$[M]^+$	342.17035	342.1700	1.07	297.09128, 265.07828, 237.08359	B
16	13.91	Coptisine	$C_{19}H_{14}NO_4$	$[M]^+$	320.09216	320.0917	1.33	318.13626, 305.11533, 292.06842, 290.09686, 277.14221, 262.15631, 249.12280	B
17	16.64	Worenine	$C_{20}H_{16}NO_4$	$[M]^+$	334.10776	334.1074	0.38	304.13062, 302.24670, 290.06900, 261.09570	B
18	14.03	Epiberberine	$C_{20}H_{18}NO_4$	$[M]^+$	336.12350	336.1230	1.39	321.12964, 320.24991, 306.12585, 292.11191, 277.98779	B
19	14.39	Columbamine	$C_{20}H_{20}NO_4$	$[M]^+$	338.13925	338.1387	1.67	323.20886, 322.30615, 308.20908, 307.35141, 294.12579, 280.21246, 279.35431	B
20	13.94	Jatrorrhizine	$C_{20}H_{20}NO_4$	$[M]^+$	338.13922	338.1387	1.58	323.19360, 322.35205, 308.19733, 294.17267, 280.33112	B
21	17.65	Palmatine	$C_{21}H_{22}NO_4$	$[M]^+$	352.15472	352.1543	1.01	337.18073, 336.41400, 322.22748, 308.14941	B
22	11.37	Berberrubine	$C_{19}H_{16}NO_4$	$[M]^+$	322.10760	322.1074	0.67	307.13080, 294.14825, 279.08707	B
23	6.57	Oblongine	$C_{19}H_{24}NO_3$	$[M]^+$	314.17535	314.1751	0.89	283.13281, 269.08923, 192.11005	B
24	11.91	Oxoberberine	$C_{20}H_{18}NO_5$	$[M]^+$	352.11862	352.1179	1.91	336.161801, 322.19922, 320.18130, 308.11249	B
25	12.67	N-Methylcanadine	$C_{21}H_{24}NO_4$	$[M]^+$	354.17111	354.1700	-0.60	339.16238, 338.33182, 336.03955, 308.23456	B
26	11.37	Groenlandicine	$C_{19}H_{16}NO_4$	$[M]^+$	322.10760	322.1074	0.67	307.13080, 292.04382, 279.08707	B
27	11.63	Demethyleneberberine	$C_{19}H_{18}NO_4$	$[M]^+$	324.12329	324.1230	0.79	309.11874, 294.11755, 280.06604, 266.15796	B

28	9.87	-	$C_{26}H_{34}NO_9$	$[M]^+$	504.22403	504.2228	2.42	206.03262, 342.21918	B
29	10.39	-	$C_{27}H_{36}NO_9$	$[M]^+$	518.24023	518.2385	3.42	206.01048, 356.25098	B
30	22.45	Acacetin	$C_{16}H_{12}O_5$	$[M+H]^+$	285.07608	285.0758	1.16	242.06549, 153.08594	B
31	25.92	Tectorigenin	$C_{16}H_{12}O_6$	$[M+H]^+$	301.07132	301.0707	2.18	286.03241, 258.36816	B
32	2.20	Danshensu	$C_9H_{10}O_5$	$[M-H]^-$	197.04535	197.0444	4.57	179.02322, 135.01001	B
33	2.24	rosmarinic acid	$C_{18}H_{16}O_8$	$[M-H]^-$	359.09763	359.0761	0.36	196.92111, 122.99706, 178.88708, 134.83539	B
34	8.69	Amurenlactone A/B	$C_{17}H_{20}O_9$	$[M-H]^-$	367.10278	367.1024	1.15	134.01590, 116.94310	B
35*	16.92	Baicalin	$C_{21}H_{18}O_{11}$	$[M-H]^-$	445.07715	445.0765	1.38	269.10269, 251.06357, 223.02805, 195.31183	C
36*	25.36	Baicalein	$C_{15}H_{10}O_5$	$[M-H]^-$	269.04514	269.0444	2.57	251.08203, 241.04578, 223.00377, 194.98622	C
37	19.37	Apigenin	$C_{15}H_{10}O_5$	$[M-H]^-$	269.04504	269.0444	2.19	251.05090, 241.04317, 223.00711, 195.08565, 169.01059	C
38	22.01	Oroxylin A 7-O-β-D-glucuronide	$C_{22}H_{20}O_{11}$	$[M-H]^-$	459.09286	459.0922	1.46	283.03824, 268.03375, 174.92964	C
39	22.50	Wogonoside	$C_{22}H_{20}O_{11}$	$[M-H]^-$	459.09305	459.0922	1.88	283.05786, 268.13113, 174.97562	C
40	24.93	Hispidulin	$C_{16}H_{12}O_6$	$[M-H]^-$	299.05560	299.0550	1.96	284.09381, 212.13605	C
41	22.24	Chrysin	$C_{15}H_{10}O_4$	$[M-H]^-$	253.05008	253.0495	2.15	225.06895, 209.17682	C
42	21.01	Wogonin	$C_{16}H_{12}O_5$	$[M-H]^-$	283.06067	283.0601	2.01	268.00586, 240.35742, 239.12109, 224.25583	C
43	25.36	Oroxylin A	$C_{16}H_{12}O_5$	$[M-H]^-$	283.06049	283.0601	1.38	268.06494, 240.94562, 224.92932, 210.99619	C
44	23.81	Norwogonin	$C_{15}H_{10}O_5$	$[M-H]^-$	269.04520	269.0444	2.79	251.08740, 241.13544, 225.01401, 223.05528, 197.04875, 169.08687	C

45	29.66	Skullcapflavone II	$C_{19}H_{18}O_8$	$[M-H]^-$	373.09229	373.0918	1.33	358.10599, 343.12820, 328.10162, 168.99115	C
46	16.98	Baicalein-7-O-glucoside	$C_{21}H_{20}O_{10}$	$[M-H]^-$	431.09790	431.0973	1.45	269.01773	C
47	19.37	Glychionide A	$C_{21}H_{18}O_{11}$	$[M-H]^-$	445.07724	445.0765	1.58	269.02609, 225.14853, 197.10564	C
48	19.90	Apigenin-7-O-β-D-glucuronide	$C_{21}H_{18}O_{11}$	$[M-H]^-$	445.07715	445.0765	1.38	269.03833, 241.02225, 175.03973	C
49	20.77	Chrysin-7-O-β-D-glucuronide	$C_{21}H_{18}O_{10}$	$[M-H]^-$	429.08234	429.0816	1.67	253.11005, 175.00330	C
50	13.01	Chrysin 8-C-glucoside	$C_{21}H_{20}O_9$	$[M-H]^-$	415.10303	415.1024	1.62	397.03436, 295.10150, 253.14240	C
51	10.76	Chrysin 6-C-arabinoside-8-C-glucoside	$C_{26}H_{28}O_{13}$	$[M-H]^-$	547.14514	547.1446	0.96	487.27631, 457.18402, 427.21051, 367.13556, 337.14490	C
52	11.72	Chrysin 6-C-glucoside 8-C-arabinoside	$C_{26}H_{28}O_{13}$	$[M-H]^-$	547.14508	547.1446	0.85	487.29669, 457.16473, 427.22186, 367.23053, 337.16998	C
53	25.09	5,7,4'-Trihydroxy-8-methoxy flavones	$C_{16}H_{12}O_6$	$[M+H]^+$	301.07016	301.0707	-1.68	286.08752	C
54	29.34	5,7-Dihydroxy-6,8-dimethoxyflavone	$C_{17}H_{14}O_6$	$[M+H]^+$	315.08698	315.0863	2.11	300.08633, 285.00574, 271.05573, 184.893194	C
55	27.96	4`,7-dihydroxyflavone	$C_{15}H_{10}O_4$	$[M-H]^-$	253.05026	253.0495	2.86	237.1283	C
56	29.67	Skullcapflavone II	$C_{19}H_{18}O_8$	$[M+H]^+$	375.10806	375.1074	1.64	360.14148, 345.09317, 342.05273, 327.12201	C
57	15.36	5,7,2',5'-tetrahydroxyflavanone-8,6'-dimethoxyflavone	$C_{17}H_{14}O_8$	$[M-H]^-$	345.06100	345.0605	1.47	330.07153, 315.16245, 287.09930	C
58	9.83	Viscidulin I	$C_{15}H_{10}O_7$	$[M-H]^-$	301.03488	301.0343	2.00	283.20975, 273.12982	C

A, Rhei Radix et Rhizoma; B, Coptidis Rhizoma; C, Scutellariae Radix

* The identification was confirmed with standard.

SUPPLEMENT TABLE 2 The identification result of components in drug plasma.

NO.	t_R/min	Identification	Compound formula	Proposal ions	Experimental (m/z)	Theoretical (m/z)	Erro ppm	MS^2 data
1	20.51	Emodin-8-O-β-D-glucoside	$C_{21}H_{20}O_{10}$	$[M-H]^-$	431.09912	431.0972732	4.284	431.09747, 269.04520, 241.05556, 225.05566
2	30.63	Emodin	$C_{15}H_{10}O_5$	$[M-H]^-$	269.04578	269.0444499	4.944	269.04507, 241.04909, 225.05550, 223.17000, 197.11797
3	28.27	Physcion	$C_{16}H_{12}O_5$	$[M-H]^-$	283.02512	283.0600999	3.074	283.06079, 269.04172, 241.04982, 240.03931, 212.04347
4	27.95	Rhein	$C_{15}H_8O_6$	$[M-H]^-$	283.02512	283.0237144	4.966	257.04520, 239.03445, 211.03969, 183.04474, 155.04980
5	29.49	Chrysophanol	$C_{15}H_{10}O_4$	$[M-H]^-$	253.05057	253.0495353	4.089	253.05019, 225.05193, 210.05298,
6	28.07	Aloe-emodin	$C_{15}H_{10}O_5$	$[M-H]^-$	269.04568	269.0444499	4.572	269.04504, 240.04211, 241.05014, 225.05530, 211.04007, 183.04475
7	10.16	Rheinanthrone	$C_{15}H_{10}O_5$	$[M-H]^-$	269.04572	269.0444499	4.721	269.04572, 225.05505, 224.06801, 197.06055, 168.96179, 150.90669
8	21.41	Chrysophanol-O-glucuronide	$C_{21}H_{18}O_{10}$	$[M-H]^-$	429.08319	429.0816231	3.652	253.05017, 225.05478,

9	20.11	Aloe-emodin-3-hydroxymethyl-O-β-D-glucopyranoside	$C_{21}H_{20}O_{10}$	$[M-H]^-$	431.09903	431.0972732	4.075	311.05624, 269.04520, 268.03842
10	20.03	Emodin-2-C-glucuronide	$C_{21}H_{18}O_{11}$	$[M-H]^-$	445.07822	445.0765377	3.78	269.04523, 253.04990, 103.00334
11	20.23	Emodin-3-O-glucuronide	$C_{21}H_{18}O_{11}$	$[M-H]^-$	445.07834	445.0765377	4.049	269.04523, 241.05048, 225.05554
12	11.61	Emodin-glucuronide	$C_{27}H_{26}O_{17}$	$[M-H]^-$	621.11035	621.1086256	2.776	621.10992, 445.07697, 269.04529, 270.04877, 225.05522
13	8.99	Rhein-8-O-glucuronide	$C_{21}H_{16}O_{12}$	$[M-H]^-$	459.05756	459.0558023	3.829	283.02438, 269.01141, 239.03442, 170.09306
14	12.76	Rhein-3-O-glucuronide	$C_{21}H_{16}O_{12}$	$[M-H]^-$	459.05746	459.0558023	3.611	283.02451, 269.04156, 239.03468, 158.84680
15	9.76	Emodin + carboxylate + hydroxylation + glucuronide	$C_{21}H_{16}O_{13}$	$[M-H]^-$	475.0524	475.0507169	3.543	299.05551, 255.06598, 227.03415, 183.02942
16	15.4	*O*-methyl-rhein	$C_{16}H_{10}O_6$	$[M-H]^-$	297.04056	297.0393645	4.025	297.04010, 253.05038, 225.05563,
17	11.34	1,8-dihydroxy-3-carboxyl-9-oxanthranol	$C_{15}H_{10}O_6$	$[M-H]^-$	285.04077	285.0393645	4.931	241.05220, 240.03795
18	17.67	8-*O*-methyl-emodin	$C_{16}H_{12}O_5$	$[M-H]^-$	283.06122	283.0601	3.957	268.03741, 240.03802, 239.03455, 211.03976
19	16.62	actyl-1,3,8-trihydroxy-6-methyl-9-oxanthranol/acetyl-1,3,8-trihydroxy-6-methyl-10-oxanthranol	$C_{17}H_{14}O_6$	$[M-H]^-$	313.07104	313.0706646	1.97	295.02432, 269.04498

20	12.35	2-hydroxy-emodin	$C_{15}H_{10}O_6$	$[M-H]^-$	285.0405	285.0393645	3.984	257.04541, 241.05046, 223.03864, 213.04552, 152.99567
21	19.34	1-*O*-methyl-emodin	$C_{16}H_{12}O_5$	$[M-H]^-$	283.06149	283.0600999	4.911	255.08080, 225.06165
22	19.46	*O*-methyl-aloe-emodin	$C_{16}H_{12}O_5$	$[M-H]^-$	283.06146	283.0600999	4.805	269.04074, 268.03732, 240.03860
23	28.56	*O*-methyl-chrysophanol	$C_{16}H_{12}O_4$	$[M-H]^-$	267.06622	267.0651853	3.874	267.06604, 239.03442
24	27.34	1,8-dihydroxy-3-methyl-9-oxanthranol/1,8-dihydroxy-3-methyl-10-oxanthranol	$C_{15}H_{12}O_4$	$[M-H]^-$	255.06625	255.0651853	4.174	225.0554
25	15.72	dehydroxy-rheinanthrone	$C_{15}H_{10}O_4$	$[M-H]^-$	253.05074	253.0495353	4.761	253.05006, 223.03986, 207.91740
26	21.37	*O*-methyl-hydroxy-rheinanthrone	$C_{16}H_{12}O_6$	$[M-H]^-$	299.05637	299.0550145	4.532	299.05637, 255.05806, 238.05876, 209.06699
27	6.63	Emodin+glucuronide+sulfate	$C_{21}H_{18}O_{14}S$	$[M-H]^-$	525.03485	525.0333522	2.853	349.00253, 269.04578, 115.00353
28	11.46	Emodin + hydroxylation+ sulfate	$C_{15}H_{10}O_9S$	$[M-H]^-$	364.99741	364.996179	3.373	285.04117, 286.04462, 241.03954
29	12.23	Emodin-1-O-sulfate	$C_{15}H_{10}O_8S$	$[M-H]^-$	349.00278	349.0012644	4.343	349.00281, 269.04575, 241.05070, 225.05501
30	12.68	Emodin + carboxylate + hydroxylation + sulfate	$C_{15}H_8O_{10}S$	$[M-H]^-$	378.97672	378.9754435	3.368	378.97678, 299.01932, 255.02971, 78.95849
31	11.99	Emodin + hydroxylation + glucuronide	$C_{21}H_{18}O_{12}$	$[M-H]^-$	461.073	461.0714523	3.357	461.07114, 285.04013, 286.04361

32	11.83	Emodin + hydroxylation+ carboxylate	$C_{15}H_8O_7$	$[M-H]^-$	299.01962	299.018629	3.314	299.01920, 255.02965, 227.03433, 211.04091
33	11.3	Emodin + methylation + hydroxylation + sulfate	$C_{16}H_{12}O_9S$	$[M-H]^-$	379.01324	379.011829	3.723	379.01160, 299.01929, 284.03250
34	24.33	Emodin + methylation + sulfate	$C_{16}H_{12}O_8S$	$[M-H]^-$	363.01855	363.0169144	4.505	363.01843, 283.06131,
35	1.97	(Gallic acid-O-CO2)-O-sulfate	$C_6H_6O_5S$	$[M-H]^-$	188.98607	188.9852204	4.495	188.98605, 109.02924
36	1.69	(Gallic acid-O+CH3)-O-sulfate	$C_8H_8O_7S$	$[M-H]^-$	246.99167	246.9906997	3.928	246.99173, 167.03488
37	19.61	Methoxy-rheinanthrone-O-glucuronide	$C_{22}H_{20}O_{11}$	$[M+H]^-$	461.10815	461.1078378	0.677	461.10815, 285.07602, 270.05243, 85.02910
38	10.28	Rhein-8-O-sulfate	$C_{15}H_8O_9S$	$[M-H]^-$	362.98203	362.9805289	4.135	283.02493, 255.03053, 239.03511, 211.03995,
39	10.45	Rhein-1-O-sulfate	$C_{15}H_8O_9S$	$[M-H]^-$	362.98224	362.9805289	4.714	283.02505, 255.03046, 239.03514, 211.04042
40	15.51	Baicalin	$C_{21}H_{18}O_{11}$	$[M-H]^-$	445.07843	445.0765377	4.252	269.04532, 197.06129, 175.02461, 113.02421
41	24.05	Baicalein	$C_{15}H_{10}O_5$	$[M-H]^-$	269.04578	269.0444499	4.944	251.16510, 241.05045, 223.03992, 195.04486, 137.09682
42	20.88	Wogonin	$C_{16}H_{12}O_5$	$[M-H]^-$	283.0614	283.0600999	4.593	268.03726, 240.04205, 239.03473, 163.00348
43	21.53	Oroxylin A	$C_{16}H_{12}O_5$	$[M-H]^-$	283.0614	283.0600999	4.593	268.03738, 241.05035, 211.03992, 165.99057
44	22.67	Chrysin	$C_{15}H_{10}O_4$	$[M-H]^-$	253.05078	253.0495353	4.919	225.05525, 209.07167,

45	20.19	Apigenin	$C_{15}H_{10}O_5$	$[M-H]^-$	269.04578	269.0444499	4.944	251.20215, 241.05032, 223.13327
46	20.11	Hispidulin	$C_{16}H_{12}O_6$	$[M-H]^-$	299.05621	299.0550145	3.997	284.03214, 212.04659,
47	21.12	Wogonoside	$C_{22}H_{20}O_{11}$	$[M-H]^-$	459.09412	459.0921878	4.209	283.06082, 268.03741, 175.02454, 113.02419
48	22.22	Norwogonin	$C_{15}H_{10}O_5$	$[M-H]^-$	269.04565	269.0444499	4.4610	250.99500, 241.01085, 225.06184, 223.04619, 197.08212, 169.08717
49	12.64	Viscidulin I	$C_{15}H_{10}O_7$	$[M-H]^-$	301.03555	301.0342791	4.2220	283.06140, 273.07733, 257.03601, 151.00377
50	17.96	Oroxylin A-5-O-glucuronide	$C_{22}H_{20}O_{11}$	$[M-H]^-$	459.09393	459.0921878	3.795	283.06073, 268.03732, 240.04124, 113.02421
51	19.62	Oroxylin A 7-O-β-D-glucuronide	$C_{22}H_{20}O_{11}$	$[M-H]^-$	459.09418	459.0921878	4.339	283.0607, 268.03735, 175.02443, 113.02416
52	9.55	Chrysin 6-C-arabinoside-8-C-glucoside	$C_{26}H_{28}O_{13}$	$[M-H]^-$	547.14594	547.1446172	2.4180	487.12488, 457.11438, 427.10339, 367.08255, 337.07187
53	9.72	Chrysin 6-C-glucoside 8-C-arabinoside	$C_{26}H_{28}O_{13}$	$[M-H]^-$	547.14618	547.1446172	2.8560	487.12497, 457.11441, 427.10358, 367.08282, 337.07205
54	20.17	5,7,4'-Trihydroxy-8-methoxy flavones	$C_{16}H_{12}O_6$	[M+H]+	301.07065	301.0706646	-0.0480	301.06934, 286.04611
55	28.25	5,7-Dihydroxy-6,8-dimethoxyflavone	$C_{17}H_{14}O_6$	[M+H]+	315.08609	315.0863146	-0.7130	300.06305, 285.03955,

56	15.54	Chrysin-5-O-glucoside	$C_{21}H_{20}O_9$	$[M+H]^+$	417.11795	417.1180086	-0.141	381.09613, 363.08450, 297.07465, 279.06442
57	11.56	Baicalein-6-O-glucose-7-O-glucuronide	$C_{27}H_{28}O_{16}$	[M+H]+	609.14532	609.1450111	0.507	271.05893
58	12.01	Baicalein-6, 7O-glucuronide	$C_{27}H_{26}O_{17}$	[M+H]+	623.12439	623.1242756	0.184	447.09103, 271.05945
59	12.41	Wogonin-5, 7-di-O-glucuronide or oroxylin A-5, 7-di-O-glucuronide	$C_{28}H_{28}O_{17}$	[M+H]+	637.14008	637.1399257	0.242	285.07495
60	11.24	Baicalein-6-O-glucuronide	$C_{21}H_{18}O_{11}$	[M+H]+	447.09244	447.0921878	0.564	271.05939
61	17.25	Dihydrochrysin	$C_{15}H_{12}O_4$	$[M+H]^+$	257.08081	257.0808354	-0.0990	257.07996
62	12.29	Apigenin-4′,7-O-diglucuronide	$C_{27}H_{26}O_{17}$	$[M+H]^+$	623.12433	623.1242756	0.087	447.09067, 271.05939
63	20.01	Viscidulin III-2′-O-β-D-glucopyranoside	$C_{23}H_{24}O_{13}$	$[M+H]^+$	509.12976	509.1289671	1.557	333.05988, 217.27946
64	23.72	Dihydroapigenin-7-O-sulfate	$C_{15}H_{12}O_8S$	$[M-H]^-$	351.01794	351.0169144	2.922	271.06387,
65	21.41	Chrysin-5-O-glucuronide	$C_{21}H_{18}O_{10}$	$[M-H]^-$	429.08319	429.0816231	3.652	253.05020, 225.05597
66	10.41	Chrysin-O-sulfate	$C_{15}H_{10}O_7S$	$[M-H]^-$	333.00766	333.0063498	3.935	253.05006, 211.04002, 135.00819
67	19.78	Baicalein-7-O-glucoside	$C_{21}H_{20}O_{10}$	$[M-H]^-$	431.09872	431.0972732	3.356	269.04062
68	10.71	Apigenin monosulfate	$C_{15}H_{10}O_8S$	$[M+H]^+$	351.01666	351.0169144	-0.725	271.05936

69	10.39	Apigenin disulfate	$C_{15}H_{10}O_{11}S_2$	$[M+H]^+$	430.97382	430.9737289	0.211	351.01581, 271.05930
70	23.58	2′,3′-Dihydroxybaicalin	$C_{21}H_{18}O_{13}$	$[M+H]^+$	479.08173	479.082017	-0.599	303.04904, 257.18976, 159.11647
71	24.13	Oroxylin A-7-O-glucoside	$C_{22}H_{22}O_{10}$	$[M-H]^-$	445.11475	445.1129232	4.104	357.09790, 225.05534, 181.06561
72	18.44	Glychionide A	$C_{21}H_{18}O_{11}$	$[M-H]^-$	445.07816	445.0765377	3.645	269.04510, 241.05023, 225.05536, 197.06042, 171.04472
73	20.72	Apigenin-7-O-β-D-glucuronide	$C_{21}H_{18}O_{11}$	$[M-H]^-$	445.07828	445.0765377	3.915	269.04510, 241.05017, 175.02451
74	24.78	7-Methoxy-baicalein	$C_{16}H_{12}O_5$	$[M-H]^-$	283.06149	283.0600999	4.911	268.03732, 163.00320
75	20.09	4'-hydroxywogonin + GluA	$C_{22}H_{20}O_{12}$	$[M+H]^+$	477.10312	477.1027524	0.77	301.07065
76	18.46	5,6-Dihydroxyflavon + GluA	$C_{21}H_{18}O_{10}$	$[M+H]^+$	431.0979	431.0972732	1.454	255.06454
77	16.55	Berberine	$C_{20}H_{18}NO_4$	$[M]^+$	336.12253	336.1230345	-1.501	320.09131, 306.07568, 292.09637
78	7.32	Magnoflorine	$C_{20}H_{24}NO_4$	$[M]^+$	342.16962	342.1699847	-1.066	297.11188, 265.08569, 237.09074
79	12.85	Coptisine	$C_{19}H_{14}NO_4$	$[M]^+$	320.09216	320.0917344	-0.67	318.07590, 292.09653, 290.08112, 277.07321, 262.08640
80	13.02	Columbamine	$C_{20}H_{20}NO_4$	$[M]^+$	338.13852	338.1386846	-0.4870	323.11432, 322.10455, 308.09045, 294.11148

81	13.47	Jatrorrhizine （Palmatine+Demethylation）	$C_{20}H_{20}NO_4$	$[M]^+$	338.13849	338.1386846	-0.5760	323.11380, 322.10608, 308.09091, 294.11145
82	16.64	Palmatine	$C_{21}H_{22}NO_4$	$[M]^+$	352.1543	352.1543347	-0.0980	337.12949, 336.12216, 322.10635, 308.12698,
83	10.51	Berberrubine	$C_{19}H_{16}NO_4$	$[M]^+$	322.10721	322.1073845	-0.5420	307.08246, 294.11099, 279.08771
84	10.87	Oxoberberine	$C_{20}H_{18}NO_5$	$[M]^+$	352.11783	352.1179491	-0.3380	336.08517, 322.06982, 322.07010, 308.09103
85	7.18	Demethylenecoptisine-di-O-glucuronide (Berberine+2 Demethylation + 2 Glucuronide conjugation)	$C_{30}H_{30}NO_{16}$	$[M]^+$	660.15588	660.1559101	-0.046	484.12091, 308.08945
86	9.01	Berberine+Reduction + Glucuronide conjugation (Jatrorrhizine-3-O-β-D-glucuronide)	$C_{26}H_{28}NO_{10}$	$[M]^+$	514.17114	514.1707724	0.715	338.13727, 323.11380
87	6.74	Didemethylation + Glucuronide conjugation	$C_{24}H_{22}NO_{10}$	$[M]^+$	484.12332	484.1238223	-1.037	308.09116
88	9.25	Berberine+Demethylation + Glucuronide conjugation	$C_{25}H_{24}NO_{10}$	$[M]^+$	498.13919	498.1394723	-0.567	322.10629, 307.08270, 225.05420
89	13.27	Dihydrocoptisine	$C_{19}H_{16}NO_4$	$[M]^+$	322.10699	322.1073845	-1.2250	322.10641, 307.08273, 279.08783
90	12.46	Berberine+Reduction + Demethylation + Hydroxylation + Glucuronide conjugation	$C_{25}H_{26}NO_{11}$	$[M]^+$	516.15027	516.150037	0.451	340.11688, 325.09332, 310.07065
91	10.06	Berberine+Reduction + Demethylation	$C_{19}H_{18}NO_4$	$[M]^+$	324.12259	324.1230345	-1.372	309.09814, 294.21521,

92	8.68	Berberine+2 Demethylation + Glucuronide conjugation	$C_{25}H_{26}NO_{10}$	$[M]^+$	500.15579	500.1551224	1.335	324.12207, 309.09845, 308.09042, 294.07462, 292.09601, 280.09601
93	10.87	Berberine+Hydroxylation	$C_{20}H_{18}NO_5$	$[M]^+$	352.11783	352.1179491	-0.338	337.09308, 336.08566, 322.06982, 308.09106,
94	13.96	Berberine+Dihydroxylation + Demethylation	$C_{19}H_{16}NO_6$	$[M]^+$	354.09695	354.0972137	-0.745	336.08548, 322.06467, 294.11142
95	11.28	Berberine+Reduction + Demethylation + Sulfate conjugation (Berberrubine+Reduction + sulfate conjugation)	$C_{19}H_{18}NO_7S$	$[M]^+$	404.079849	404.07974	-0.27	324.12189, 309.09863
96	13.75	Berberine+Dihydroxylation	$C_{20}H_{18}NO_6$	$[M]^+$	368.11276	368.1128637	-0.282	336.08228, 320.05377
97	13.41	Palmatine+Hydroxylation + Demethylation +Glucuronide conjugation	$C_{26}H_{28}NO_{11}$	$[M]^+$	530.16479	530.165687	-1.692	354.13251, 339.10898, 324.08578, 310.10474
98	23.22	Palmatine+Reduction + Hydroxylation + Methylation	$C_{22}H_{26}NO_5$	$[M]^+$	384.18076	384.1805494	0.548	366.09634, 338.10220,
